# Looking for the crystal ball in unscheduled care: a systematic literature review of the forecasting process

**DOI:** 10.1007/s10729-025-09711-z

**Published:** 2025-05-23

**Authors:** Mingzhe Shi, Bahman Rostami-Tabar, Daniel Gartner

**Affiliations:** 1 Cardiff Business School, Cardiff University, Cardiff, Wales United Kingdom; 2 School of Mathematics, Cardiff University, Cardiff, Wales United Kingdom; 3 National Health Service (NHS), Aneurin Bevan University Health Board, Caerleon, United Kingdom

**Keywords:** Forecasting, Healthcare, Unscheduled care, Time series, Regression, Classification

## Abstract

The ability to accurately forecast unscheduled care needs is of paramount importance for decision making in healthcare operations, ensuring a continuous and high-quality level of care. In this work, we provide a literature review of 156 research articles of forecasting applications with special focus on care services that are not scheduled in advance such as emergency departments. Our paper presents two key contributions. Firstly, we propose a novel framework designed to characterize the application of forecasting process across various unplanned healthcare services. Our taxonomy facilitates the detection, decomposition, and categorization of forecasting processes, enhancing the understanding of their deployment in different unscheduled care settings. Secondly, we conduct a comprehensive literature review based on a systematic search, critically analyzing the state of forecasting research in unscheduled care services and identifying key research gaps. We explore forecasting problems in depth, examining their purpose, the various methodologies used, the rigor used in generating and evaluating forecasts, and the reproducibility of results, all within the context of the proposed framework. By consolidating the current state of the art, this paper provides valuable insights to both healthcare professionals and academics regarding the effective application of forecasting in unscheduled care services. Finally, it serves as a roadmap for identifying major research gaps and outlines an agenda for future investigations.

**Supplementary Information:**

The online version contains supplementary material available at 10.1007/s10729-025-09711-z.

## Introduction

Unscheduled care, often referred to as unplanned or emergency care, encompasses vital healthcare services that are not scheduled in advance and are essential for addressing immediate or unforeseen health issues. These critical services remain accessible around the clock, prioritizing patients’ needs beyond the confines of routine healthcare services [1–3]. In recent years, the escalating number and diversity of services providing unscheduled care have prompted policymakers and researchers to perceive these services as a cohesive system (see Section 3.1 for a detailed description). Given its uncertainty and unpredictable nature, this system has brought unique challenges to management, planning, and decision-making [4]. These challenges may include overcrowding, putting extra pressure on healthcare professionals, missing waiting time targets [5], and ultimately deteriorating the quality of service. Applying forecasting algorithms is one of the possible practical approaches to inform better resource capacity planning that may help overcome these challenges mentioned above [6].

Forecasting is the process of making predictions about future outcomes based on historical data. This process involves several interconnected stages that can be repeated to improve accuracy and reliability, known as the forecasting process [7]. In recent years, it has been frequently applied in unscheduled care services, among other applications [8], such as emergency department attendance [9, 10], patients’ length of stay [11], and resource requirements [12, 13].

While there are various literature reviews on healthcare analytics [3, 14–17], there are several limitations that motivate our systematic literature review, which are summarized below: i) many of these reviews focus on specific elements of unplanned care, such as emergency departments. In contrast, our systematic review takes a broad approach, examining unscheduled care in its entirety; ii) past reviews have only focused on the forecasting technique, ignoring the process involved in making reliable forecasts. In our research, we prioritize reviewing the whole forecasting process in the literature, acknowledging its importance in providing actionable insights to healthcare decision-makers; iii) there is a significant gap in the previous reviews surrounding the evaluation of forecasting uncertainty. This component is critical for making informed decisions; however, it is often overlooked. Our work fills this gap by highlighting the uncertainty considerations with forecasts in the published literature; iv) the field of forecasting in healthcare continues to evolve, driven by advances in modern modeling approaches. Recent developments are covered in the current review; and v) there has been no prior review that integrates various aspects of unscheduled care, planning and decision-making, and the forecasting process (workflow) in a comprehensive framework. This is critical since components of a forecasting process are always determined by the planning and decision-making and the environment (i.e., various elements of unscheduled care services) in which they operate.

In this review, we introduce a framework that allows us to investigate the use of the forecasting process at multiple decision-making levels-operational, tactical, and strategic-within unscheduled care services, offering a comprehensive perspective beyond existing studies. Unlike previous studies that only looked at forecasting methods [18] or resource capacity [19], our framework emphasizes the connection between the forecasting process and specific unscheduled care settings, ensuring that decision-making processes are informed by a rigorous process across different care dimensions.

Using a systematic literature review, we offer a more transparent, thorough, and complete evaluation compared to standard narrative reviews. Furthermore, our review addresses the following research question: ”Drawing from existing research, how is the forecasting process in unscheduled care settings being addressed to support planning and decision-making at the operational, tactical, and strategic levels?” Addressing this question helps to identify current practices in forecasting within unscheduled care, highlight gaps in the literature, and provide recommendations for improving practice and guiding future research efforts.

We have three main objectives: (1) compile and synthesize current literature on forecasting in unscheduled care, based on the framework presented in Fig. 2, (2) investigate critical elements of the forecasting process, such as purposes, evaluating quality, and reproducibility, and (3) highlight gaps in the literature and propose an agenda for future studies. Our framework serves as a valuable resource for researchers by highlighting gaps in the current literature and offering a roadmap for future studies. For healthcare practitioners, it enhances their understanding of the key elements of the forecasting process necessary to develop a rigorous process tailored to different decision-making levels within unscheduled care.

The remainder of this paper is organized as follows: The next section provides a description of the search process used to identify relevant articles for the review. In Section 3, our conceptual framework is provided, focusing on care services, planning, and decision levels, as well as the forecasting process. Section 4 then examines and categorizes each of the discovered papers from the literature search to give an overview of forecasting purposes and variables, forecasting methods, probabilistic forecasting, evaluating quality, as well as reproducibility of research. Section 5 explores disparities and challenges when forecasting for unscheduled care services. Section 6 concludes with a summary followed by detailed characteristics of publications in the Appendix. Finally, our supplementary file provides an additional breakdown of the literature.

**Fig. 1:**
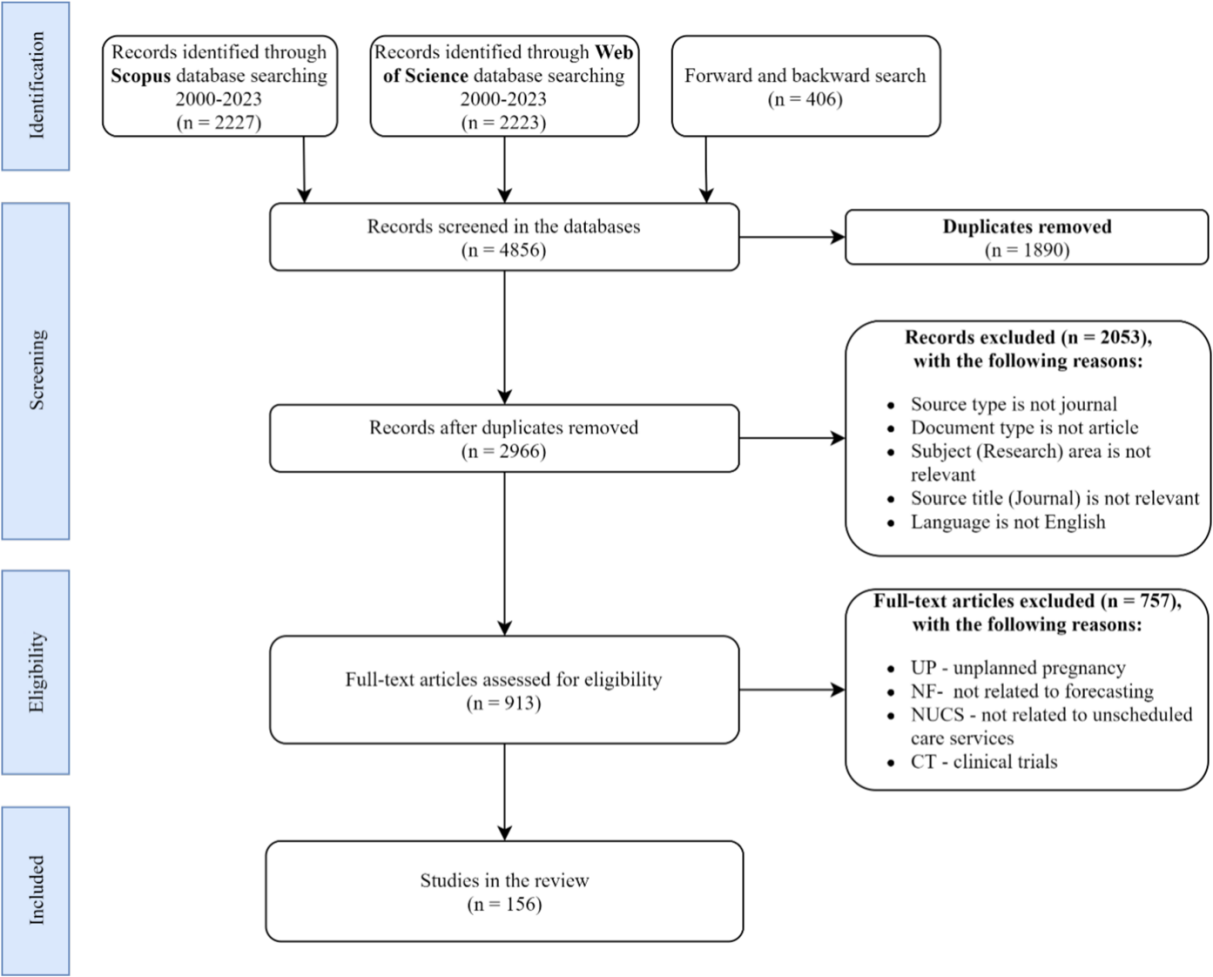
PRISMA diagram detailing the search process [21]

## Search methodology

A systematic literature review was conducted using electronic databases on Scopus as well as the Web of Science to search for English publications. The choice of Scopus and Web of Science was driven by their comprehensive coverage of relevant databases and journals, which are essential for the interdisciplinary nature of our literature review on forecasting in unscheduled care services. These databases are renowned for their extensive indexing of operational research (OR) and management science (MS) journals, making them ideal for our aim to review literature pertinent to forecasting within unscheduled care services. The search process is summarized in Fig. 1. To locate the most relevant articles in the final sample for the review, search terms provided in Table 1 were used while applying a combination of these terms using the “AND” and “OR” Boolean operators. The keywords were chosen based on preliminary scans of existing literature reviews. [3, 14–16, 20].

**Table 1:** Search term to identify the literature base set

Search Terms	Boolean Operator
Health*	AND
Admission	OR
Ambulatory	OR
Emergency	OR
Emergency call	OR
Emergency department	OR
Forecast*	AND
Health care	OR
Home care	OR
Intensive care unit	OR
Predict*	AND
Primary care	OR
Social care	OR
Surgical	OR
Telemedicine	OR
Unplanned	OR
Unscheduled	AND
Urgent care cent*	OR

This research concentrates on operational research (OR) and management science (MS) techniques that support and rationalize resource capacity planning. Based on demand forecasting, these methods provide optimization tools for designing the unscheduled care delivery processes [2]. We extensively reviewed papers pertinent to forecasting within unscheduled care services, published in OR/MS Journals from 2000 to June 2023. The timeframe between 2000 and June 2023 was selected to capture the most significant advancements in forecasting methodologies and notable changes in healthcare policies that have influenced the landscape of unscheduled care. The OR/MS journals selected in this article are obtained from the Journal Citation Report (JCR) by Clarivate Analytics in 2022.

The screening stage is demonstrated in Fig. 1 by applying a structured approach detailed using a PRISMA diagram [21]. Our search process utilized a combination of automated and manual strategies to ensure both comprehensiveness and precision. Automated searches were conducted in Scopus and Web of Science using predefined keywords and Boolean operators, allowing us to gather a broad set of relevant articles quickly. Subsequently, a manual review of these results was performed to identify the most pertinent studies, including a forward and backward search to capture additional relevant publications not initially identified.

**Fig. 2:**
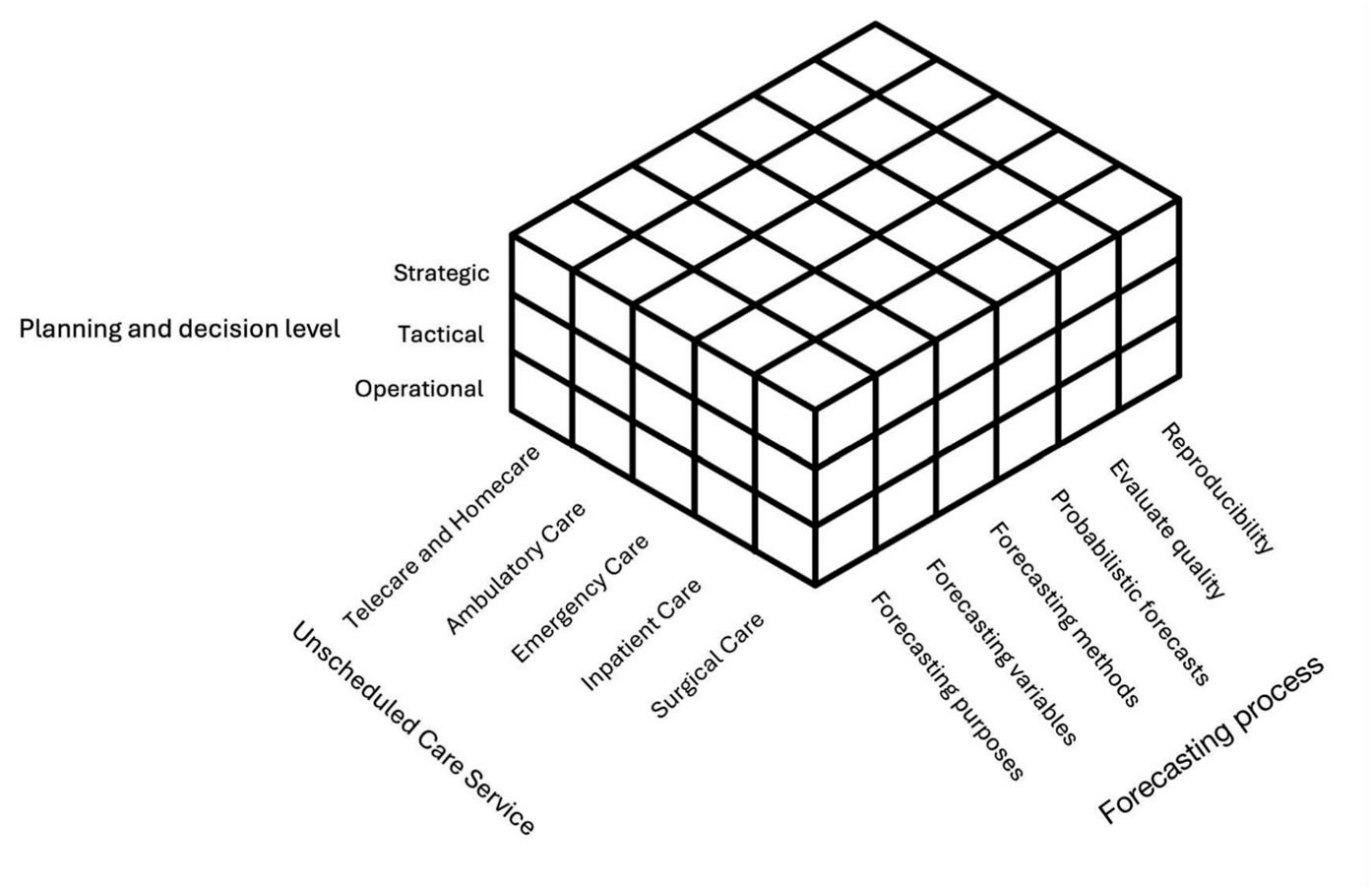
Forecasting in unscheduled care services: a framework

Figure 1 also reveals that the identification process includes 2227 and 2223 records for Scopus and the Web of Science, respectively. Another 406 publications were retrieved through a forward and backward search. The initial electronic database search produced 4856 records. After the removal of 1890 duplicates, a total of 2966 articles of potential relevance remained. Then, 2053 articles were subsequently excluded because the source type is not a journal or the journal theme is irrelevant, such as chemistry, earth, and planetary sciences. We further refined our selection of the remaining 913 articles by excluding those primarily focused on clinical outcomes, such as evaluating specific factors leading to unplanned readmissions. This exclusion was necessary to ensure that our review remains aligned with the objectives of this literature review, focusing on system-level forecasting rather than individual patient outcomes. By doing so, we aim to provide insights that are more directly applicable to resource planning and management in unscheduled care settings, which is crucial for operational, tactical, and strategic decision-making. As a result of this process, a total of 156 articles were considered relevant and non-redundant, which are reviewed in detail in the remainder of this paper.

## Forecasting in unscheduled care services: a conceptual framework

To explore the application of forecasting in unscheduled care services, we suggest a framework with the following main dimensions: i) unscheduled care service, including telecare and home care, ambulatory care, emergency care, inpatient care, and surgical care; ii) planning and decision levels, including operational, tactical, and strategic levels; and iii) forecasting process, including forecasting purposes, forecasting variables, forecasting methods, probabilistic forecasts, evaluate quality, and reproducibility.

These three dimensions are combined to create a novel framework to assist the forecasting application in unscheduled care services, as illustrated in Fig. 2. This framework provides a structured overview of forecasting in unscheduled care and enables researchers and practitioners to identify relevant approaches and best practices for their specific operational decisions and care settings. In the following subsections, we will discuss the three main dimensions of the framework.

### Unscheduled care service

Unscheduled care services are organized into five categories based on several factors, including urgency, resource intensity, and patient outcome potential, as discussed in Webb [3] and Hulshof et al. [2]:

1. **Telecare and Homecare Services**: These services include a variety of remote support services, allowing patients to remain independent for an extended period or coordinate services for a patient at their home. Telecare and homecare services are provided by service providers such as emergency call services, telemedicine services, housing care, etc. [2].
2. **Ambulatory Care Services**: In addition to providing scheduled care, ambulatory care services often treat patients with acute illnesses and minor injuries that are not anticipated to be life-threatening. In the context of unscheduled care, we focus on the unpredictable aspects of ambulatory care, such as same-day appointments for urgent medical needs. Examples of ambulatory care services are general practitioners (GP), minor injury units (MIU), and walk-in centers (WIC) [2, 3].
3. **Emergency Care Services**: Responsible for evaluating and treating urgent and emergent medical issues, such as those caused by accidents, trauma, acute sickness, poisoning, or catastrophes. Emergency medical treatment may be delivered in a hospital setting or locations outside of the medical institution. Examples include hospital emergency rooms, ambulances, and trauma centers [3].
4. **Inpatient Care Services**: Defined as care provided to a patient who has been officially admitted for treatment and will remain in the hospital for a minimum of one night. This level of service includes accommodations to meet patients’ special needs, such as hospital admissions or general nursing wards [2, 3].
5. **Surgical Care Services**: These include surgical operations for the correction of deformities and defects, the treatment of injuries, and the diagnosis and treatment of specific illnesses. Surgical care services include operating theatres, intensive care units (ICU), and others [3].

### Planning and decision level

Healthcare organizations make planning and control choices to create and run the healthcare delivery process. It requires integrated long-, medium-, and short-term planning and decision-making across several management functions [6]. We provide a taxonomy for the planning and decision levels, which includes the following three classes:

1. **Long-term strategic planning and decision-making**: It entails an evaluation of the whole unscheduled care system at a strategic level. Strategic planning typically ranges from 1 to 10 years.

2. **Medium-term tactical planning and decision-making**: It outlines the techniques the organization intends to use to accomplish the objectives mentioned in the strategic plan [2]. Tactical planning usually has a time horizon of less than a year and occurs monthly.

3. **Short-term operational planning and decision-making**: These are often called one-time or continuing plans [22] or decisions. From a temporal perspective, operational planning occurs on a sub-daily, daily, or weekly basis.

### Forecasting process

We consider several essential elements of the forecasting process [7], which are outlined in the sections below:

1. **Forecasting Purposes**: There is at least one or multiple reasons why a forecast might be required for unscheduled care services. These are generally determined by identifying planning and decisions, essentially what the forecast will inform. This is what we refer to as forecasting purposes.

2. **Forecasting Variables**: Forecasting variables refer to the output of the forecasting model we intend to forecast. This might also be called outcome, response variable, or target. The forecast variable is generally used as an input (alongside other potential inputs) to the decision-making process. The purpose of forecasting dictates the forecast variable.

3. **Forecasting Methods**: A well-organized classification system for forecasting techniques may facilitate the selection of the most suitable forecasting method. In this study, we present a classification of forecasting methods, which might aid in the comprehension and organization of the approaches used in the construction of a forecasting system. The forecasting category employed in this review is based on Petropoulos et al. [8], which illustrates the typologies and methodologies used in each research. The forecasting methods are classified as:

i) *Statistical and econometric models* (e.g., exponential smoothing (ES), auto-regressive integrated moving average, logistic regression): These models are highly relevant in situations where there is a wealth of historical data, and the relationships between variables are relatively stable over time. In healthcare, these methods are particularly suited for forecasting patient admissions, disease progression trends, or inventory needs, where past data can reliably inform future decisions. For example, ARIMA or exponential smoothing is well-suited for forecasting patient flow in ED over time, allowing hospitals to identify cyclical patterns in patient demand, especially during flu seasons or pandemics. The ability of these models to incorporate and adjust for past trends makes them highly reliable for short-term forecasting in stable environments.

ii) *Variable and model selection* (e.g., lasso regression, elastic-net): These methods are valuable when dealing with complex datasets where identifying key predictive factors is crucial for informed decision-making. In healthcare, selecting the most relevant variables can enhance model interpretability and reduce overfitting, ensuring that predictions are both accurate and actionable. Methods like LASSO regression and Elastic-Net automatically shrink less important variables toward zero, effectively performing both regularization and feature selection.

**Fig. 3:**
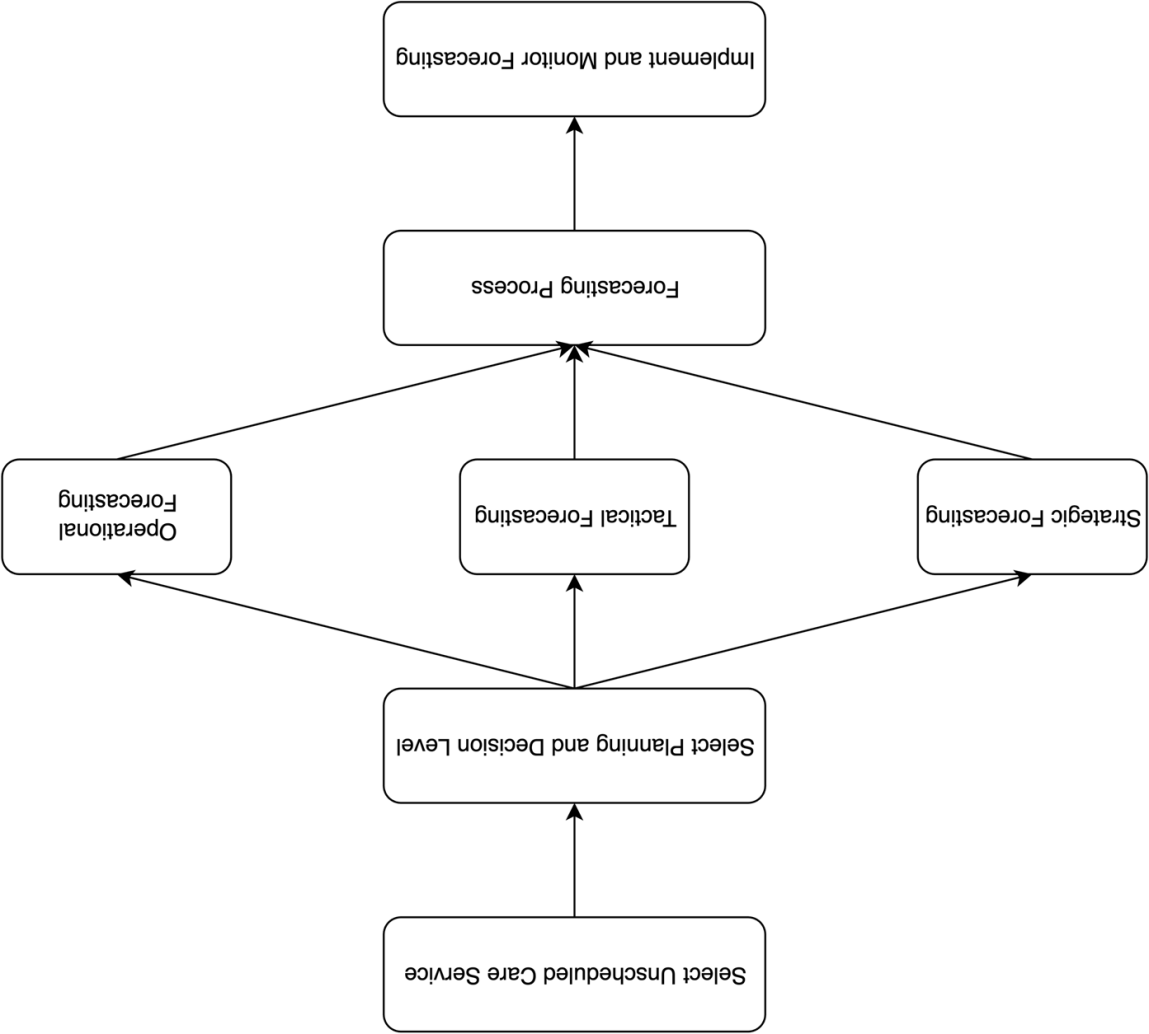
Step-by-step guide for practitioners

iii) *Data-driven methods* (e.g., artificial neural networks (ANN), support vector machines (SVM), bayesian forecasting): Machine learning-based models such as artificial neural networks (ANN) and decision trees (DT) are particularly powerful in healthcare contexts where data is abundant but relationships between variables are complex and nonlinear. These models are often used for predicting disease outbreaks, optimizing treatment plans, and making individualized patient predictions. For instance, Random Forests and LSTM models are particularly useful for forecasting patient outcomes by analyzing the vast amount of clinical data, such as lab results, patient histories, and real-time monitoring data. These models excel in uncovering patterns that may not be immediately apparent, enabling more accurate predictions of disease progression. These models help clinicians make personalized treatment decisions and improve patient care outcomes, especially in dynamic and high-pressure environments like intensive care units.

iv) *Other forecasting models* (e.g., Judgmental forecasting and simulation methods): Judgmental forecasting and simulation methods are more suited to scenarios where human expertise and intuition are critical, or when there is limited or uncertain data. In healthcare, these methods are beneficial when forecasting the impact of rare events or managing resource planning in highly unpredictable situations, such as during pandemics or in emergency preparedness. Simulation models, like Monte Carlo simulations, can help healthcare managers to evaluate different policy scenarios and make informed decisions about capacity planning or crisis management.

An alternative to the above classification might be based on the types of amounts forecasted, which may help in the selection of an appropriate approach for a certain application. For example, some forecasting techniques may be suitable for durations such as patient wait times or hospital length of stay, while others may be tailored to quantities such as admissions or call volumes.

4. **Probabilistic Forecasts**: Generating and communicating probabilistic forecasts is essential for acknowledging uncertainty in predictions. Rather than providing a single-point estimate, probabilistic forecasts offer a range of potential outcomes along with their associated probabilities. This approach allows decision-makers to better understand and manage uncertainty, leading to more informed and flexible decision-making. We assess whether the research incorporates probabilistic forecasting methods to account for and represent uncertainty in its forecasts.

5. **Evaluate Quality**: We assess the forecasts’ quality by analyzing several factors that ensure a rigorous implementation of the forecast quality evaluation process. These factors include: i) Benchmark: is a benchmark method included for comparison? ii) Out-of-sample evaluation: is out-of-sample used to examine accuracy? iii) Cross-validation: is cross-validation used to report accuracy? iv) Error metrics: what error metrics are used to quantify the model’s performance? v) Uncertainty metrics: is forecast uncertainty being measured and reported? vi) Business utility metrics: does the research assess the business value of the forecast in a practical setting?

6. **Reproducibility**: Ensuring that forecasts are reproducible is vital for transparency and validation. This involves clear documentation of forecasting methods, data sources, and assumptions, allowing other researchers to replicate the study and verify its findings.

To effectively make use of the proposed framework for forecasting in unscheduled care services, practitioners are guided through a structured process as depicted in Fig. 3. Healthcare providers should begin by selecting the specific unplanned care service type, followed by the level of planning and decision-making involved. They then need to determine different elements of the forecasting process. For instance, an ambulatory care clinic could apply the approach for day-to-day decisions using short-term forecasting techniques to anticipate daily patient numbers, thus enhancing staff planning. On the other hand, a hospital might utilize it for long-term strategic planning to assess the necessity for new emergency department facilities. The approach helps in choosing appropriate elements in the forecasting process for the service type and decision-making level. This structured approach ensures that the forecasting process is not only theoretically robust but also practically applicable to real-world decision-making in unscheduled healthcare settings, addressing the specific needs and operational goals of different care providers.

## Research analysis within the framework

This section presents the analysis of the literature review, focusing on various elements of the framework illustrated in Fig. 2. Subsection 4.1 outlines the characteristics of the studies, considering the three dimensions of the framework. Given the fundamental importance of forecasting process, Sections 4.2 to 4.6 highlight findings specifically related to the dimensions of forecasting process.

### Article characteristics

When analyzing the frequency of papers across various unscheduled care services categories, we found that emergency care services receive the most attention. This is not surprising because urgent and emergency care go hand in hand. The role of emergency care as one of the primary gateways into hospitals also contributes to its prominence. Ambulatory and surgical care receive the least attention in the unscheduled care system. Another observation is that 33 studies apply their prediction to two care dimensions, while only eight articles [23–30] address three or more care dimensions.

The limited representation of surgical care in research literature could be due to several reasons. One possible explanation is that these services are often perceived as urgent or time-critical compared to emergency care, leading to a lower priority when it comes to forecasting efforts in these areas. Moreover, data availability and quality challenges can create obstacles for researchers aiming to build forecasting models for surgical care services. These services typically involve a range of patient conditions, treatment paths, and outcomes, making it challenging to gather and standardize the required data for forecasting purposes. Additionally, the fragmented nature of healthcare systems and the lack of integrated data systems might impede reliable data collection throughout the care process, especially for services beyond hospital settings. Overcoming these data-related barriers and promoting collaboration among healthcare stakeholders, researchers, and policymakers will be essential in broadening the scope of forecasting studies to cover an array of unscheduled care services.

We assess the frequency of publications associated with each category of forecasting methods. Among these, statistical and econometric models are the most often used, followed by data-driven models, and variable and model selection methods. There is a limited amount of research involving judgmental forecasting when dealing with forecasting unplanned activities in healthcare. There were 36 publications identified as using multiple classes of approaches. Thirty-two of the studies use two approaches, while only one utilize three approaches [31]. The majority of these papers use statistical and econometric models along with one other method, demonstrating that multiple clusters of forecasting methods could be used to solve one problem. These findings highlight the potential for integrating different forecasting approaches to enhance predictive accuracy, providing valuable insights for decision-makers and practitioners seeking to improve forecasting strategies in complex and uncertain environments.

While most of the current literature focuses on statistical and machine learning models, there is indeed potential for judgmental forecasting to play a more significant role, especially in situations where expert intuition and real-time decision-making are critical. Given the unpredictability of unplanned care, incorporating expert judgment into forecasting models could complement data-driven approaches, improving accuracy in scenarios where historical data alone may not fully capture the complexities of care demand. Future research could explore how to effectively integrate judgmental methods with existing models to enhance the forecasting process.

Given that most forecasting tasks may require using many variables, which can be time-consuming and expensive, it is unsurprising that that many authors consider using variable and model selection methods. Benefits of using this method include delivering variables more rapidly and cost-effectively by lowering training time, facilitating data visualization, and providing a greater overall understanding of the forecasting procedure. It also aids in selecting a model with few variables by eliminating unnecessary factors that render the model more accurate and easy to comprehend [32]. However, it is essential to scrutinize the widespread adoption of variable and model selection methods critically. Their popularity, while suggestive of utility, may not fully capture their efficacy relative to other methodologies. For instance, while these algorithms simplify models by focusing on key variables, they may overlook complex interactions that more advanced techniques could capture. Therefore, we propose a reflective examination of whether the current preference for variable and model selection methods is truly performance-driven or a result of unchallenged acceptance.

**Fig. 4:**
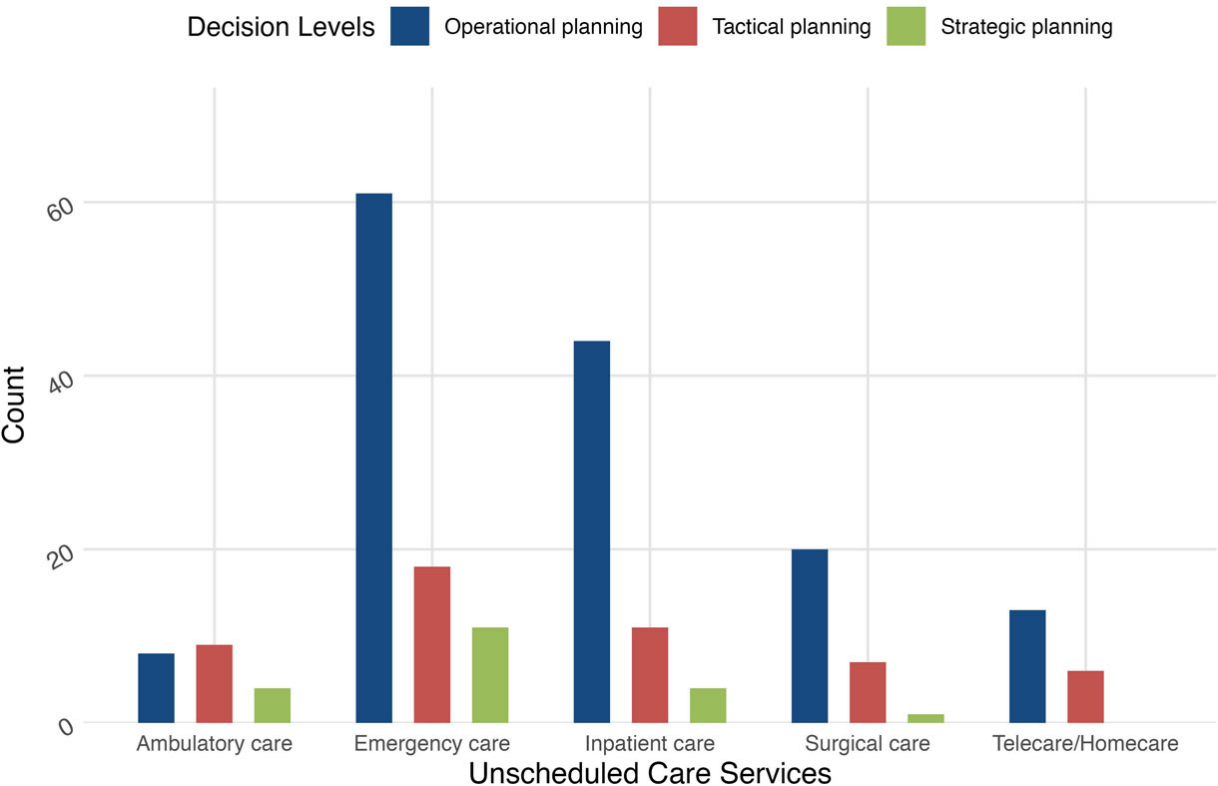
Interaction between unscheduled care services spectrum and planning and decision levels

Providing decision-makers with information on the various planning levels in different services might assist them in evaluating and altering the organization’s direction in response to a changing environment. The connection between the unscheduled care services and planning and decision levels is shown in Fig. 4.

**Fig. 5:**
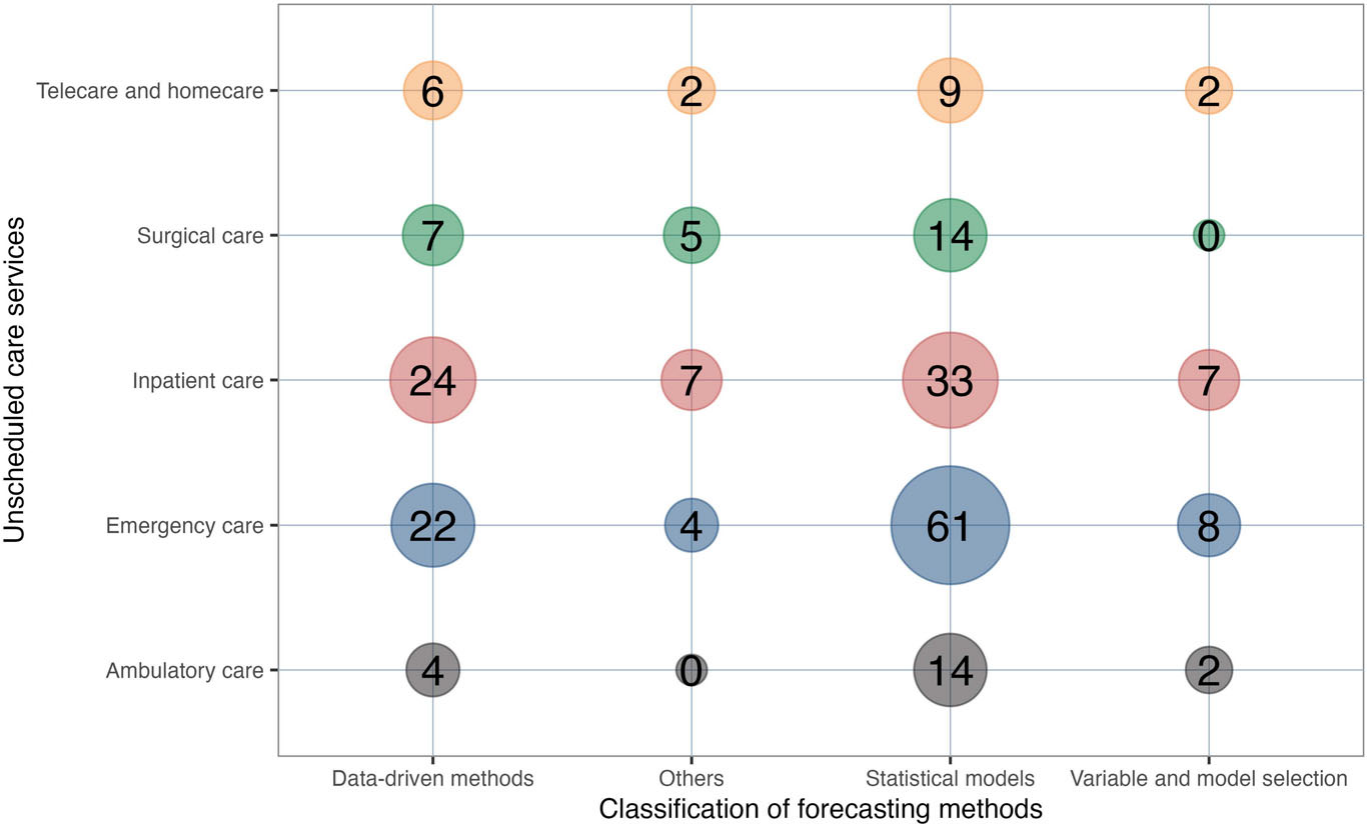
Interaction between unscheduled care services spectrum and forecasting methods

Due to the scarcity of long- and medium-term forecasting research, the three levels of planning decisions are not equally distributed, with operational planning being more prevalent than the other two decision levels. There is a lack of forecasting studies on strategic planning in telecare and surgical care. This reveals a research gap in the long-term planning of these two services.

Operational environments change among various unscheduled care levels, and forecasting strategies should be adapted accordingly. For example, telecare receives more aggregated data in their daily operation, and forecasting strategies that require more individual data, such as variable and model selection methods, may not be suitable for this environment. When managers select a forecasting method, they are unsure whether it is suitable for their operational environment. The interaction between the forecasting methods and unscheduled care levels is considered and displayed in Fig. 5 to provide a realistic means of selecting an appropriate forecasting approach.

**Fig. 6:**
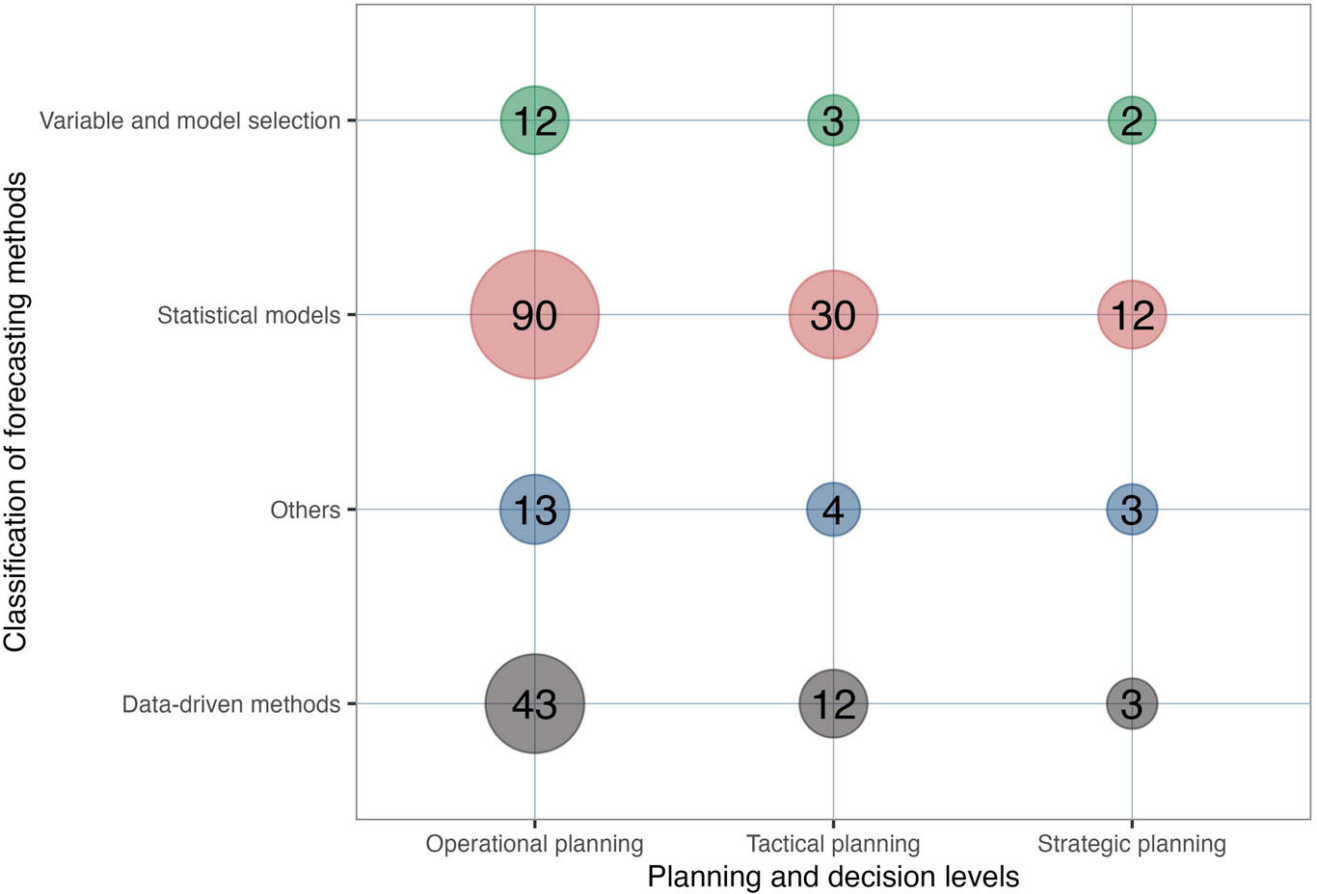
Interaction between planning and decision levels and forecasting methods

The figure indicates that statistical and econometric models and data-driven forecasting models are applied more frequently in inpatient care. More research utilizes statistical and economic models for emergency care services compared to inpatient care and telecare. In statistical and econometric models, logistic regression and the ARIMA models are the most frequently used forecasting techniques, whereas Lasso regression is the most frequently used forecasting method in variable and model selection methods. The artificial neural network (ANN) is the most frequently utilized approach in data-driven models. This result is not surprising because of their outstanding ability to model non-linear and complex relationships between statistical variables used for forecasting. These findings provide useful insights for decision-makers and practitioners in healthcare, highlighting the importance of selecting appropriate forecasting methods based on the specific needs of different care settings to improve planning and resource allocation.

Finding a link between decision levels and forecasting techniques can give forecasters a direction for selecting the appropriate forecasting method based on their planning horizons. It can reduce system error if an adapted model is used [33]. Figure 6 considers the interaction between planning and decision levels and forecasting methods. This figure shows that short-term statistical and econometric models are most frequently used across all three planning and decision levels. The interaction between operational planning and statistical and econometric models was the most observed, and multivariate logistic regressions were the most used method in all three decision levels. This can be explained as this method is the most frequently used by academics.

To further enhance the decision-making focus of our framework, Supplementary Tables 6-15 categorize the forecasting process related to the different planning and decision-making levels (strategic, tactical, and operational). The tables offer healthcare decision-makers concrete examples from the literature, connecting the planning decisions with the forecasting purposes. Also, the table serves as a practical tool to help decision-makers navigate the complexities of unscheduled care and identify appropriate actions for their operational needs.

**Fig. 7:**
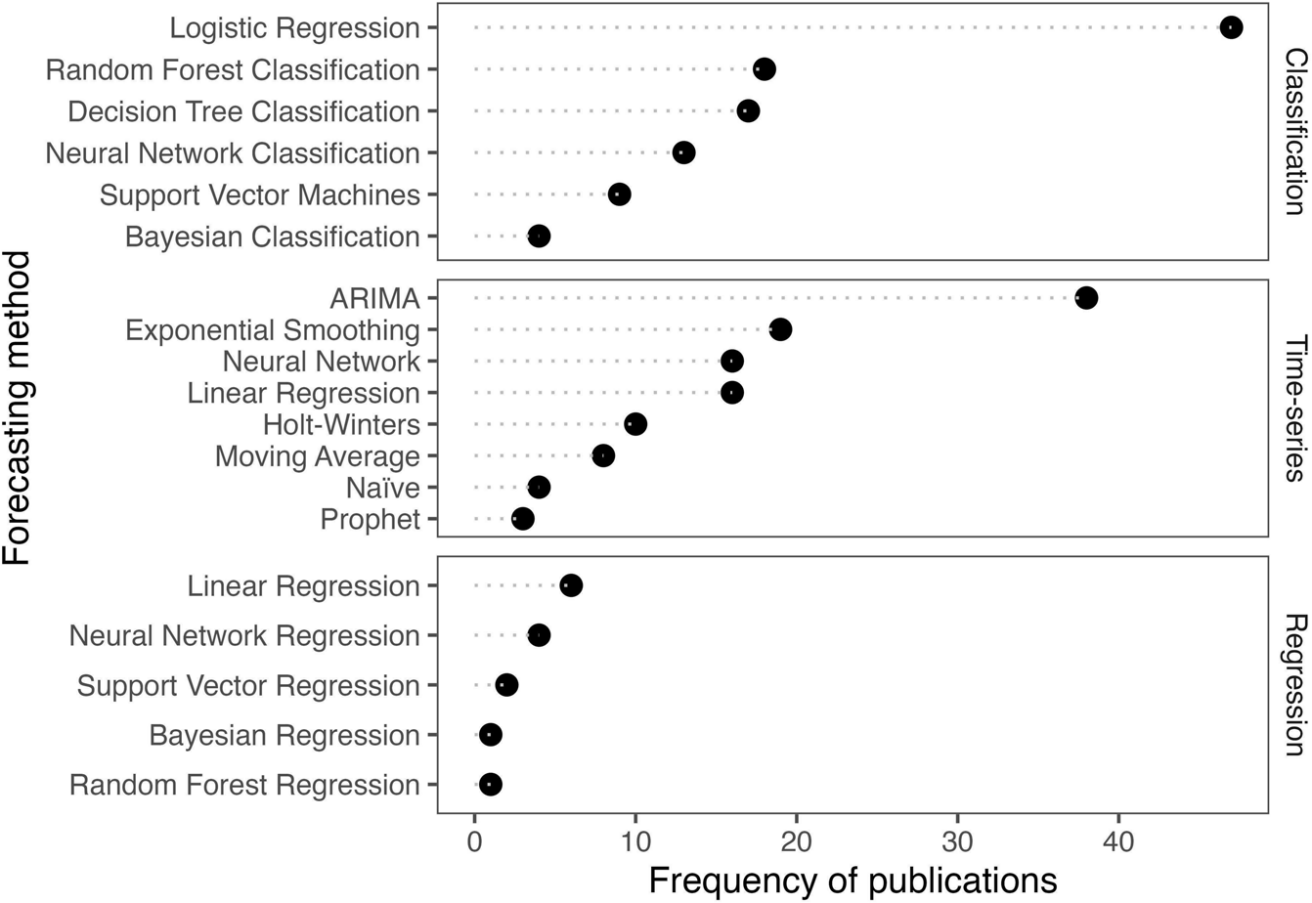
Forecasting methods used in the unscheduled care literature

### Forecasting purposes and variables

Our research finds that the main purpose of producing a forecast is to identify unplanned readmissions and optimize resource allocation and staffing. This shows that researchers are more interested in discovering patients’ readmission trends, such as risk indicators for patient visits and admission schedules. There is little research examining these two purposes. We also identify which variables are commonly used in the forecasting process. Analyzing forecasting variables allows researchers to appreciate the most recent study trends and distribution.

Our analysis indicates that ”Readmission,” ”ED attendance,” and ”Emergency call volume” are among the most utilized forecast variables among researchers. However, these variables are not all modeled using the same methods. For example, ”Readmission” forecasting tends to rely heavily on machine learning models such as neural networks, while ”ED attendance” and ”Emergency call volume” often use time series models like ARIMA or exponential smoothing. This highlights a range of modeling approaches that vary depending on the specific variable being forecasted.

### Forecasting methods

In this section, we review various forecasting methods found in the literature and classify them as regression problems, time series, and classification problems. This categorization can aid researchers in examining relevant papers during the forecasting process. In our literature review, there are more articles addressing classification problems ($n = 68$) than regression problems ($n = 12$) and time-series problems ($n = 64$).

Figure 7 displays the breakdown of the research’s classification, regression, and time-series methods. It reveals that more than half of the studies rely on logistic regression when performing classification modeling. Linear regression is the most considered approach for regression problems. When analyzing time-series problems, the ARIMA method ($n = 38$) is the most frequently used method, followed by exponential smoothing ($n = 19$) and neural networks ($n = 16$). Given that a statistical model like ARIMA is effective for regression and time series problems but ineffective for classification, this is not a surprising result. While variable and model selection methods are effective at dealing with categorization challenges, it is difficult to determine the optimal number of input variables [8].

### Probabilistic forecasts

Producing the probability distribution for the forecast variable allows the decision-maker to examine the underlying uncertainty. It presents a range of possible future values within which there is a probability that the outcome will occur. We assess whether probabilistic forecasting methods are employed in the research to represent and manage uncertainty in forecasts in this section. Probabilistic forecasting is not commonly used in this area. Some articles discuss only prediction intervals or quantiles, which is different from providing the entire forecast density, i.e., probabilistic forecasts. 41% of studies ($n = 64/156$) generate prediction intervals or quantiles, and less than 6% of studies ($n = 9/156$) consider using probabilistic forecasts evaluation metrics. Details of the articles analyzed during the literature review are provided in Supplementary Tables 6 to 15.

### Evaluate quality

Evaluating the quality of forecasting models is crucial for ensuring their reliability in decision-making. This section focuses on how models are validated, including the use of cross-validation, out-of-sample evaluation, benchmarking, error metrics, uncertainty metrics, and business utility metrics, which are essential for assessing performance beyond historical data.

A forecasting model is trustworthy and deemed fit to act robustly in future scenarios if its accuracy has been examined using out-of-sample data through cross-validation and the study includes the result of simple benchmarks. Our analysis shows that 76% of studies ($n = 121/156$) employ both in-sample forecast validation and out-of-sample forecast evaluation. Furthermore, 60% of research articles ($n = 93/156$) do not examine forecast accuracy using cross-validation, with model performance likely to be overestimated. 63% of the examined research ($n = 98/156$) considered using benchmark models of forecasting to compare their model performance. From that, 64% of articles ($n = 63/98$) employ benchmark models to address regression and time-series problems, and only 36% ($n = 35/98$) do so when solving classification problems.

**Fig. 8:**
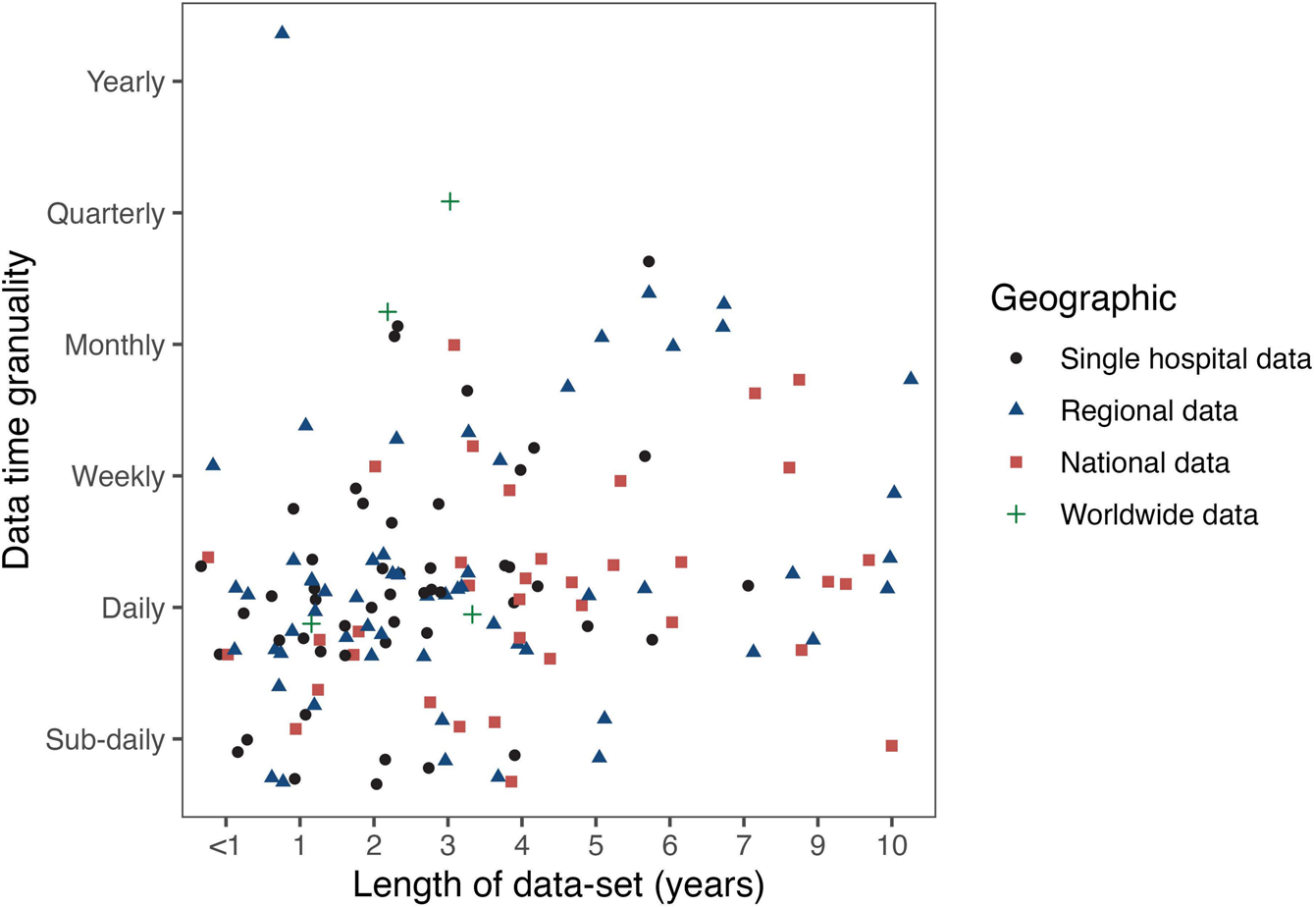
Scatter plot illustrating datasets utilized in the literature, categorized by length and time granularity

The area under the receiver operating characteristic (AUC-ROC curve) is the most often used assessment metric ($n = 54$), which is suitable for classification problems, followed by mean absolute percentage error (MAPE) ($n = 32$) and root mean square error (RMSE) ($n = 28$) to assess point forecasts. 41% of studies ($n = 64/156$) report the evaluation of prediction intervals and less than 8% of studies ($n = 12/156$) consider using the pinball score to assess the performance of probabilistic forecasts.

A forecast should not be regarded as an ultimate objective but rather as an input that must be integrated into a decision support tool to be most effective. Linking forecasts to their utility can aid decision-makers in comprehending the advantages of forecasting and adjusting their strategy accordingly. In this research, we also examine whether the utility of forecasts, such as reporting cost, waiting time, or staff utilization, are evaluated and reported. We find that there are only 9% of papers ($n = 14/156$) evaluating their forecasting utility. Four papers investigated patient waiting time and length of stay [34–37], six evaluated staff rotation [30, 38–42], and nine evaluated resource use and cost [25, 26, 36, 39, 42–46]. Four papers evaluated more than one forecasting utility [30, 36, 39, 42]. Most papers lack solid measurement of their forecasting model utility, demonstrating a disconnect between academic and practical environments.

### Reproducibility

Reproducibility and transparency are crucial for generating trustworthy forecasts that are implemented in practice to inform better decision-making. Sharing data and codes helps researchers to reproduce studies and enhance the scientific credibility of published works. Our results show that only 5% of studies ($n = 8/156$) share data and codes aiming at reproducibility and transparency. 15% of studies ($n = 24/156$) share only data, and 8% of studies ($n = 13/156$) share only codes, whereas most studies cannot be reproduced due to the lack of published data or code. One explanation for this might be the sensitivity of data in healthcare, as most researchers are required by hospitals not to expose their datasets to protect patients’ privacy.

Figure 8 explores how the data has been used when applying forecasting to unscheduled care services. 24% of papers ($n = 38/156$) use one year or less than a year-old data set. Approximately half of the studies ($n = 71/156$) utilize data spanning two to five years, while 15% of papers ($n = 23/156$) use data spanning more than five years. Literature reviews and other papers ($n = 24/156$), which do not specify a data period, are classified as using unknown datasets. When we examine the time granularity of data utilized in studies, we see that the bulk of research uses daily data. Still, long-term time granularity, such as quarterly and annual datasets, is uncommon.

We also explore the spatial applicability of the data and divide it into four main groups: i) single hospital data, including data from a single hospital; ii) regional data, including data from multiple hospitals in the same region; iii) national data, including multiple hospitals for one country, which is applicable to generating forecasting models for the whole country; and iv) worldwide data, including data sources from different countries. Twelve papers do not specify how they obtained the data and are classified as unspecified. As seen in Fig. 8, most studies are based on data from a single hospital or a specific regional area. Only 35 articles, however, use national or worldwide data.

## Discussion

Recent research underscores the growing adoption of forecasting in unscheduled care services due to their potential to inform decision-making and improve service quality. However, challenges persist. Inequality within the healthcare system, encompassing horizontal and vertical aspects, presents complexities. Our analysis reveals substantial inequalities at both geographic and service levels. Horizontal inequality surfaces in imbalanced research projects across geographic locations, dataset lengths, and forecasting horizons, particularly in underrepresented economies or communities. Vertical inequality is evident in excessive focus on emergency care, neglecting ambulatory care. Addressing these disparities is essential for improving the forecasting process, the corresponding decision-making, and resource allocation. In the following sections, we explore these topics in greater detail and examine the challenges involved in designing rigorous forecasting processes.

A forecast is not an end by itself; it is produced to inform better decisions. If decision-makers refuse to use forecasts due to a lack of trust, they are useless no matter how accurate they are. Therefore, having a rigorous process in place is crucial to produce forecasts. Furthermore, examining the accuracy of forecasts could help build trust and reliability in a health service.

Our analysis reveals that the need for developing a rigorous process in this area remains largely unmet, and the lack of thorough forecasting research implementation remains a major limitation in informing planning and decisions in practice. To improve the trustworthiness of the forecasting process, any forecasting task should: i) produce and evaluate not only point forecasts (i.e. single number) but also the entire forecast distribution that is essential to manage risk; ii) evaluate the accuracy of out-of-sample data using cross-validation; iii) report both point forecast error metrics and uncertainty metrics to acknowledge uncertainty in forecasts; iv) when comparing forecast accuracy among multiple approaches, including a benchmark method to demonstrate the forecast added value, either the current applied forecast model or a simple model known in the area.

The lack of research reproducibility is another major concern contributing to the lack of trust in forecasting and modeling unscheduled care activities. Reproducible research will not only increase transparency and consequently build trust but also increase the possibility of an impact in the real world by providing problem owners or policymakers with applicable tools or insights. Sharing code and data is a critical step in improving the reproducibility and research quality of prediction modeling. However, data sharing in the context of health services research might be difficult. The primary reason for this is that under strong confidentiality and privacy agreements, many routinely obtained health data are only released to researchers for scientific reasons with the authority of the company that created them [47], making external validation and individual-participant data meta-analysis challenging to accomplish. We recommend employing open-source software such as R, Python, and Julia, and using modern open-source scientific and technical publishing tools such as Quarto to increase reproducibility and transparency in this area.

Complex models may be developed, but they typically have little impact in the real world because they do not provide problem owners or policymakers with actionable insights [48]. This problem is related to how forecasts should be communicated to decision-makers in terms of not only the forecast error but also the business value that matters to a decision-maker. The quality of forecasts is regularly evaluated using error metrics. However, common error measures fail to consider a forecast’s usefulness in making better decisions. Therefore, we need to extend traditional error measures to account for forecast utility in decision-making and report the implications of different forecasts on metrics such as costs, service levels, and waiting times. The proposed measures require simulating the decisions that depend on the forecasts using computer models. These models take any forecast input, simulate the decision process, and evaluate the quality of the final decision based on utilities that are important to the business. Our results show that only a few studies consider the business value of forecasts in addition to the forecast error. Therefore, more research should be devoted to forecasting and decision-making optimization in unscheduled care services.

Although studies have identified various forecasting purposes, the research in academic literature predominantly has local or restricted applicability. Our analysis shows that most of the forecast variables and their purposes focus on readmission, emergency department arrivals, and calls, which are all related to the demand at the entry point of the care system. Researchers should broaden their scope of inquiry by investigating other forecast variables in the system. Therefore, we should forecast not only the total aggregated demand but also more granular information such as demographics and clinical characteristics of patients entering the service, their waiting time in each service, types of service required, and length of their stay. The forecasting process could be enhanced through the integration of variables and information drawn from a broad spectrum of unscheduled care service categories [31, 49]. For example, studies in recent years have focused on using triage information from ED to predict hospital admissions [49–51]. Future studies could forecast patients’ demand for emergency care services using telecare data, such as calls received in a clinical desk service or the number of verified incidents. This may require future researchers to collect data from several departments within a hospital or mix data from multiple service providers’ databases.

Our findings indicate that although most published studies focus on statistical and machine learning models, the application of judgmental forecasting methods in unscheduled care services has not yet been explored. It is critical that we blend academic theory with practical application. During this procedure, researchers should enlist the assistance of other professionals to care for unplanned patients and incorporate their theories into a predictive model. For example, researchers could investigate the consequences of high-acuity patients by consulting with specialists in this field. Moreover, most studies use separate individual models to forecast the need for services; there is a scope to investigate the application of global models [52] in this area.

The application of the forecasting model across multiple care dimensions presents several challenges. Firstly, each dimension operates under different constraints and priorities, making a one-size-fits-all model impractical. Methodologically, creating a model that is general enough to span multiple dimensions yet specific enough to be actionable is complex. Data limitations also play a role; each care dimension generates distinct types of data at varying granularities and frequencies. This heterogeneity complicates the development of a universal model that can accurately forecast across all dimensions without significant customization. Furthermore, the integration of data across services for comprehensive modeling is often hampered by interoperability issues, privacy concerns, and the sheer volume of data to be processed. These challenges suggest the need for a tailored approach to forecasting for each care dimension and the given planning and decision level, with interdisciplinary collaboration to create cohesive models that can adapt to the intricacies of each service type.

To address these challenges, one of the key strengths of the proposed framework is its ability to adapt to varying decision-level needs across unscheduled care settings, from ambulatory care to emergency services. For example, in operational environments like urgent care centers, where short-term forecasting is critical, data-driven methods like Artificial Neural Networks (ANN) can be employed to predict patient volumes on a daily basis, helping to optimize staffing and reduce wait times. For strategic planning, such as hospital expansion decisions, more complex time-series models that combine outputs from models with potential judgmental information from experts can be useful to forecast long-term horizons in emergency department visits, supporting resource allocation and infrastructure development. This adaptability ensures that the framework is not just an academic exercise but a practical tool that can guide decision-making at all levels of unscheduled care management. The combination of method selection with clear performance assessments also addresses one of the key challenges highlighted in the literature: the need for actionable, data-driven insights that can directly impact patient outcomes and service efficiency.

Among models relying on quantitative data, most studies use statistical models and data-driven methods. It is advised that future research develop forecasting methods using ensemble models, such as combining models like data-driven models and statistical models, which might improve forecast accuracy. Additionally, While little research has been conducted on Bayesian forecasting, we suggest that the application of this approach should be investigated in future studies. Given its ability to incorporate prior knowledge and dynamically update predictions, Bayesian forecasting has the potential to improve predictive accuracy, particularly in complex and uncertain environments. Expanding research in this area could lead to more robust forecasting models that enhance decision-making across various domains. Our results also indicate that most of the forecasting research provides point forecasts (i.e., a single number). Researchers should consider using probabilistic forecasting (i.e., forecast distribution or density) to better help planners and decision-makers manage risk, where the tails (i.e., low probability outcomes) are of interest rather than just normal situations.

Producing probabilistic forecasts is critical for planning and decision-making and enhances risk management in unscheduled care by offering a comprehensive view of potential outcomes and associated probabilities. Unlike deterministic forecasts, probabilistic models acknowledge uncertainty, aiding resource allocation and contingency planning. However, the transition to probabilistic forecasting comes with significant computational and interpretive challenges. From a computational perspective, probabilistic models often require more advanced techniques, increased data storage and processing capabilities, and may impact computational time and resources. Interpreting and communicating probabilistic forecasts to decision-makers accustomed to point estimates can also be challenging, necessitating strategies for effectively visualizing and explaining uncertainty. Organizations must also consider the potential trade-offs, such as increased model complexity, the need for specialized expertise, and the risk of information overload when presenting multiple scenarios. To successfully implement probabilistic forecasting, we recommend conducting pilot studies, investing in staff training, and establishing clear guidelines for model validation and performance monitoring. By carefully navigating these challenges and adopting a phased approach, organizations can realize the benefits of probabilistic forecasting in enhancing decision-making and resource allocation in unscheduled care settings.

Our observation indicates that the research has primarily focused on generating forecasts for a single level of planning and decision horizons, for a given unscheduled care service. With advances in data collection technologies, data in unscheduled care services are often collected at a disaggregate level that could be transformed into multiple levels of hierarchical and grouping structures (such as national, regional, health board, hospital, priority, type of injury, age group) and temporal (such as sub-daily, daily, weekly) levels. Given that planning and decisions are relevant at operational, tactical, and strategic levels, forecasting is also required to be produced at multiple levels of cross-sectional and temporal levels. This is also critical for coordinating activities across different departments and functions, where forecasting the same variables is required across different services. While these approaches have been recently developed in the forecasting research [53] and been applied in various domains [54, 55], this has been completely ignored in forecasting for unscheduled care services.

### Limitations

This literature review comes with some limitations that are described in this section. To begin with, planning and decision levels are primarily classified according to the forecasting horizons used in studies. However, no specific decision horizon length is stated for any of the hierarchical planning stages since they are determined by the application’s unique characteristics. For example, the emergency room’s planning horizons are estimated to be shorter than those of a nursing home’s long-stay ward in a particular area.

Meanwhile, it is important to note that the literature review does not include master’s and doctoral theses, as well as book chapters, and only looks at research conducted in English. Additionally, certain forecasting methods used in practice may never have been considered for publication, and their results are thus irrelevant to our analysis. Finally, the articles included in this literature review were mainly retrieved from Web of Science and Scopus.

## Conclusions

This review aims to explore how forecasting is applied in unscheduled care services. The review thoroughly evaluates 156 articles on forecasting for unscheduled care services. It presents a comprehensive framework of unscheduled care forecasting that could be used as a guide for healthcare practitioners and researchers interested in this area. The framework classifies unscheduled care services into multiple categories and examines their interactions with planning & decision levels and forecasting processes. All articles are summarized, and discussions are provided according to publication trends, forecasting purposes and variables, planning and decision levels, forecasting methods, model performance evaluation, and study design rigors to give readers a better scope of recent developments in unscheduled care forecasting.

Considering the projected growth in demand for unplanned healthcare services, using forecasting models to forecast the future is becoming increasingly significant. This article outlined some important actions for both professionals and researchers in this area, which include:

1. **Making the forecasting process a priority**: Instead of focusing solely on forecasting methods, healthcare organizations should prioritize the entire forecasting process and establish dedicated teams to engage in the entire process from identifying purpose to communicating forecast and reproducible practices. While integrating real-time data in the forecasting process, a culture of data-driven decision-making should be fostered. Furthermore, training to enhance staff proficiency in forecasting should be a priority.

2. **Shift from point estimates to probabilistic forecasts**: Probabilistic forecasts improve risk management in unscheduled care by offering a range of potential outcomes and associated probabilities, acknowledging uncertainty to aid resource allocation and contingency planning. However, this transition brings computational and interpretive challenges, requiring advanced techniques, more data storage, and processing power. Communicating these forecasts to decision-makers used to point estimates can also be difficult, necessitating clear visualization strategies. Organizations must balance increased model complexity and the risk of information overload. To implement probabilistic forecasting, we recommend pilot studies, staff training, and clear guidelines for model validation. Software tools such as Python libraries (e.g., *statsmodels*, *Prophet*, *TensorFlow Probability*) and R packages (e.g., *fable*) provide practical solutions for navigating these complexities. Following this step-by-step approach can help organizations improve decision-making and resource management in unscheduled care settings.

3. **Strategic focus and methodology enhancement**: Future research and practical applications should explore the integration of advanced optimization techniques with forecasting models to support effective planning & decision-making. This may involve the development of hybrid models that combine predictive analytics with simulation and optimization methods, as well as the creation of decision support tools that enable healthcare managers to evaluate the impact of different resource allocation strategies under various demand scenarios.

4. **Granular forecasting**: Future applications should prioritize a more granular approach to forecasting within unscheduled care, focusing on variables such as patient conditions, demographic factors, and specific service demands. Rather than relying solely on broad categories like total admissions or overall resource use, forecasting models should aim to predict the needs of specific patient subgroups or conditions. This level of detail would enable healthcare providers to better anticipate fluctuations in demand and optimize resource allocation. For instance, forecasting the needs of patients with chronic conditions, or those at higher risk of readmission, could help reduce bottlenecks in emergency departments or improve staffing for specialized care. Additionally, granular forecasting could incorporate real-time data inputs, such as patient vitals or symptom onset patterns, which could significantly enhance the responsiveness of the healthcare system to emerging trends. As a result, more tailored, proactive interventions could be designed, improving patient outcomes and overall system efficiency. Future studies should explore the potential of machine learning and artificial intelligence to handle such granular data inputs effectively and to scale these insights across different unscheduled care settings.

5. **Forecasting for multiple planning levels**: Healthcare practitioners and researchers should explore the application of temporal and hierarchical aggregation in forecasting unscheduled care services. This approach enables the generation of forecasts at multiple temporal or cross-sectional levels rather than focusing on a single level, by leveraging and combining data across various levels. In addition to improving forecast accuracy, it may also enhance coordination between different departments.

6. **Transparency and reproducibility**: For forecasting theory and practice to contribute meaningfully to unscheduled healthcare management, transparency and reproducibility must be key priorities. Researchers and analysts should ensure that their methodologies, data, and results are clearly documented and made accessible to others in the field. This would allow independent verification of findings and enable other researchers to build upon previous work, thus reinforcing the credibility of forecasting models and outcomes. Additionally, reproducible studies that openly share their datasets and algorithms not only bolster confidence in their conclusions but also facilitate the widespread adoption of effective forecasting tools across different healthcare systems. Transparency also plays a vital role in bridging the gap between academic research and practical implementation. By openly addressing the limitations and uncertainties inherent in forecasting models, researchers can foster a more collaborative approach with healthcare practitioners, ensuring that models are both scientifically robust and practically relevant. Future research should focus on creating open-access platforms and fostering collaborations that promote reproducibility, which will drive forward innovation and establish a foundation of trust in the forecasting tools applied in unscheduled care.

Due to the increasing demand for unscheduled care services, this review underscores the importance of forecasting in navigating through this changing trend. To efficiently take advantage of forecasting abilities, we suggest a workable plan supported by our framework. First, an introduction of domain-specific analytics teams within healthcare institutions should be encouraged to drive the forecasting process to ensure that they both stand scientifically and are tailored towards unique dynamics in each care category. Secondly, probabilistic forecasting models should be adopted more quickly so that decision-makers can use ranges of potential outcomes when allocating resources in uncertain situations. Thirdly, traditional metrics must be surpassed as operational impacts become the main way to assess forecast quality by bringing judgmental opinions into forecasts and using advanced ensemble methods to improve predictions.

Several areas require further research in forecasting for unscheduled care services. One key area is the integration of real-time data into forecasting models; future studies should explore how data such as live patient admissions, emergency call volumes, and telemedicine interactions can improve forecasting accuracy and responsiveness in dynamic care environments. Additionally, hybrid models that combine statistical and machine learning techniques with optimization offer the potential for enhanced decision-making in complex and uncertain unscheduled care settings [17]. While our primary focus is on the forecasting process rather than method classification, we acknowledge that certain forecasting approaches might be better suited to specific decision-making levels and unscheduled care settings. Future research could explore which classes of methods are most appropriate for different decision levels and care settings, providing further insights into their applicability. Moreover, more research is required to focus on granular forecasting at the patient level to gain deeper insights into what strategies work best under different conditions. While most research has focused on operational planning, there is a clear need to develop innovative long-term forecasting methodologies [56] to inform strategic planning and decision-making. Moreover, effective integration of probabilistic forecasting into the planning horizon, along with an evaluation of its impact on planning and decision-making, is crucial. Finally, building trust among researchers through transparency and reproducibility in publications will foster collaboration, driving innovation and progress in the field.

## Supplementary Information

Below is the link to the electronic supplementary material.

Supplementary file 1 (pdf 231 KB)

## Data Availability

The datasets generated and analyzed through this literature review are available at: https://github.com/Shimingzhe123/Literature-review-data.git
