# Supplementary material for: Looking for the crystal ball in unscheduled care: a systematic literature review of the forecasting process
Source: Health Care Manag Sci. 2025 May 23;28(3):548–64. doi: 10.1007/s10729-025-09711-z (PMC12535502; doi:10.1007/s10729-025-09711-z)
Supplement: Supplementary file 1 — (pdf 231 KB) [file 10729_2025_9711_MOESM1_ESM.pdf]

1 Supplementary Materials

Table 1: References for Planning and Decision Levels

Operational planning	[3–9, 11, 13–15, 17–19, 21–23, 25–32, 34–39, 43–47, 49, 51–56, 58–60, 62–64, 66–71, 73–76, 80, 83–97, 99, 101, 102, 104, 105, 107–114, 117–126, 128–136, 140, 142–151, 155, 156]
Tactical planning	[2, 4, 6, 9, 11, 14, 17, 18, 20, 23, 26, 27, 33, 41, 42, 60, 62, 66, 67, 72, 77, 78, 82, 85, 93, 100, 110–112, 118, 137, 140, 150, 152, 154, 156]
Strategic planning	[1, 2, 4, 17, 21, 24, 26, 50, 60–62, 65, 72, 81, 98, 104, 106, 127, 137, 139, 148, 152]

Table 2: References for Unscheduled Care Levels

Telecare and homecare	[6, 11, 28, 34, 65, 85, 99, 100, 102, 110, 111, 117, 124, 134, 146, 147]
Ambulatory care	[21, 38, 40–42, 48, 50, 61, 64–66, 86, 87, 100, 105, 106, 110, 111, 116, 127, 147, 152]
Emergency care	[1, 4–6, 8–10, 12–14, 17, 18, 20, 22, 24, 26, 27, 29–31, 33, 37, 39, 42, 46, 48, 49, 51, 51, 53, 54, 56–59, 63, 64, 66, 67, 69–72, 74, 76, 79–82, 84, 86, 88, 92, 97, 100, 101, 104, 106, 108–111, 113, 114, 116, 118, 125, 128, 129, 134–137, 139–141, 143, 144, 148, 149, 151, 152, 155]
Inpatient care	[2, 3, 7, 10, 15, 19, 23, 25, 32, 35, 39, 42–45, 47, 49, 52, 54, 55, 60, 62, 68, 69, 71, 75, 77, 78, 84, 86, 90, 93, 94, 96, 98, 101, 105, 106, 108, 110, 111, 113, 119, 120, 122, 123, 125, 126, 130, 131, 133, 138, 145, 153, 154]
Surgical care	[2, 18, 25, 36, 41, 60, 73, 83, 84, 86, 87, 89–91, 95, 103, 107, 108, 110–112, 121, 132, 142, 150, 156]

Table 3: References for Forecasting Methods

Data-driven model	[2, 5, 7, 10, 11, 15, 19, 20, 22, 25, 35–37, 39, 42, 44, 52–56, 58, 67, 68, 73, 75, 77, 78, 85, 86, 88, 89, 92, 96, 99–101, 106, 121, 122, 124, 128, 131, 134, 148, 150, 152, 154]
Statistical and econometric model	[1, 2, 4, 6, 8, 9, 13, 14, 17, 20, 24, 27, 29, 31, 33–35, 37, 41, 45, 47, 63, 64, 66–72, 74, 76, 78, 83, 88, 91, 97, 99, 102, 104, 106, 110–112, 117–120, 129, 135–137, 143, 144, 146, 149, 155, 156]
Variable selection algorithms and regression models	[3, 8–10, 13, 18, 21–23, 26, 30, 32, 38, 39, 42–44, 46, 49, 50, 52, 54, 58, 59, 61, 65, 79, 80, 82, 86–90, 92–94, 96, 98, 99, 101, 105, 107–109, 113, 114, 117, 118, 122, 123, 125–127, 130, 134, 139, 140, 142, 147–149, 151, 154]
Other model	[2, 28, 47, 60, 62, 81, 84, 95, 102, 108, 132, 133, 145]

Table 4: Summary of Previous Literature Reviews

Literature review	[12, 16, 40, 48, 57, 103, 115, 116, 138, 141, 153]

Table 5: Acronyms used in the paper

**Acronym**	**Full Form**	**Acronym**	**Full Form**
AIC	Akaike Information Criterion	ANN	Artificial Neural Network
AM	Additive Model	APDR	Average Peak Demand Rate
ARIMA	Auto-Regressive Integrated Moving Average	ATE	Approximate t-based errors
AUC-ROC	Area Under the Receiver Operating Characteristic Curve	AUPRC	Area Under Precision-Recall Curve
BBM	Bivariate Bayesian model	BIC	Bayesian Information Criterion
BN	Bayesian Network	BOR	Bayesian ordinal regression
BS	Brier Score	CART	Classification and Regression Trees
CI	Confidence Interval	CNN	Convolutional Neural Network
CR	Coverage Rate	CRPS	Continuous Ranked Probability Score
CT	Classification Tree	Catboost	Categorical Boosting
Chi-squared	Chi-squared Statistic	DPA	Demand pattern analysis
DRL	Decision Rule Learners	DT	Decision Tree
DTR	Decision Tree Regressor	ED	Emergency Department
EHR	Electronic Health Records	EMG	Empirical Mode Decomposition
EN	Elastic Net	ER	Error Rate
ES	Energy Score	ETS	Exponential Smoothing
FME	Fluid Model Estimators	FTS	Fuzzy Time Series Forecasting Model
G-mean	Geometric Mean	GAA	Generic Analytical Approach
GAMs	Generalized Additive Models	GARMA	Generalized Auto-Regressive Moving Average
GBM	Gradient Boosting Machine	GBT	Gradient Boosted Tree Model
GEE	Generalized Estimating Equations	GLM	Generalized Linear Model
GP	General Practitioners	GPEMR	General Practitioner Electronic Medical Records
GRU	Gated Recurrent Unit	HLS	Hosmer-Lemeshow statistic
HRS	Hazard Ratio for Survival	HW	Holt-Winters
ILL	Intermittent Local Level	JCR	Journal Citation Report
JF	Judgemental Forecasting	KNN	K-Nearest Neighbors
LASSO	Least Absolute Shrinkage and Selection Operation	LOS	Length of Stay

Acronym	Full Form	Acronym	Full Form
LSTM	Long Short-Term Memory	LWR	Locally Weighted Regression
LightGBM	Light Gradient Boosting Machine	LogR	Logistic Regression
MA	Moving Average	MAD	Mean Absolute Deviation
MAE	Mean Absolute Error	MAPE	Mean Absolute Percentage Error
MARS	Multivariate Adaptive Regression Splines	MASE	Mean Absolute Scaled Error
MC	Monte Carlo Simulation	ME	Mean Error
MEDAE	Median Absolute Error	MIU	Minor Injury Units
MLP	Multi-Layer Perceptron	MPR	Multivariate Probit Regression
MRAE	Mean Relative Absolute Error	MRE	Mean Relative Error
MS	Management Science	MSARIMA	Multiplicative Seasonal Auto-Regressive Integrated Moving Average
MSE	Mean Square Error	NAR	Nonlinear Auto-Regressive
NBR	Negative Binomial Regression	NBT	Naive Bayes Technique
NLLSR	Nonlinear Least Squares Regression	NLP	Natural Language Processing
NPV	Negative Predictive Value	OR	Operational Research
OT	Optimal Trees with Parallel Splits	P-value	Probability Value
PA	Predictive Analytics	PAR	Poisson Autoregressive
PBE	Percentage Bias Error	PPV	Positive Predictive Value
PR	Poisson Regression	PRISMA	Preferred Reporting Items for Systematic Reviews and Meta-Analyses
PS	Pinball Score	PerS	Percentage Score
Q-LASSO	Quantile Least Absolute Shrinkage and Selection Operator	QM	Queuing Models
QR	Quantile Regression	RBFN	Radial Basis Function Network
REML	Restricted Maximum Likelihood	RF	Random Forest
RMAE	Relative Mean Absolute Error	RMAP	Relative Mean Absolute Percentage
RMSE	Root Mean Square Error	RMSPE	Root Mean Squared Percentage Error
ROC	Receiver Operating Characteristic Curve	RPS	Rank Probability Score
RR	Ridge Regression	RW	Random Walk
SAPE	Symmetric Absolute Percent Error	SARIMA	Seasonal Auto-Regressive Integrated Moving Average
SBC	Schwartz Bayesian Criterion	SD	Standard Deviation

Acronym	Full Form	Acronym	Full Form
SE	Standard Error	SLR	Simple Linear Regression
SM	Simulation Method	SMHW	Simple Moving Histogram Weighted
SSA	Singular Spectrum Analysis	STLF	Short-Term Load Forecasting
SVM	Support Vector Machine	TBATS	Trigonometric, Box-Cox, ARMA, Trend, and Seasonal Components
V-ARMA	Vector Auto-Regressive Moving Average	VAR	Vector Autoregression
WAIC	Watanabe-Akaike Information Criteria	WAPE	Weighted Absolute Percentage Error
WD	Wasserstein Distance	WIC	Walk-in Centre
WMAPE	Weighted Mean Absolute Percentage Error	WMSE	Weighted Mean Square Error
WS	Weighted Sum	Xgboost	Extreme Gradient Boosting

Table 6: Characteristics of publications in telecare and homecare services (a)

References	Planning and decision level(s)	Forecasting purposes	Forecasting variables	Forecasting methods	Probabilistic forecasts
Al-Azzani et al. [6]	Operational,Tactical	To minimize response times to emergency calls	Emergency call volume	ARIMA,HW,LR,SSA	✗
Argiento et al. [11]	Operational,Tactical	To optimize resources allocation and staff	Nurses visits	BB	✓
Brown et al. [28]	Operational	To minimize response times to emergency calls	Emergency call volume	DPA	✓
Channouf et al. [34]	Operational	To minimize response times to emergency calls	Emergency call volume	ARIMA	✗
Inouye et al. [65]	Strategic	To detect unplanned patient readmission	Patient re-admission	LogR	✓
Lanzarone et al. [85]	Operational,Tactical	To optimize resources allocation and staff	Nurses visits	MC	✗
Martin et al. [99]	Operational	To minimize response times to emergency calls	Ambulance demand	MLP,DT,KNN,HW,ARIMA	✗
Martinsson and Gustafsson [100]	Tactical	To estimate the effects of telephone nursing	Healthcare utilizations	BOR	✓
Matteson et al. [102]	Operational	To minimize response times to emergency calls	Emergency call volume	GAMs	✗
Ordu et al. [110]	Operational,Tactical	To optimize resources allocation and staff	Service demand	ARIMA,ETS,LR,STLF	✗
Ordu et al. [111]	Operational,Tactical	To optimize resources allocation and staff	Service demand	SLR,ETS,STLF,ARIMA	✗
Rostami-Tabar and Rendon-Sanchez [117]	Operational	To improve the ability of hospital to tackle pandemic	COVID-19 confirmed cases	LR,ARIMA,ETS,Naive,Prophet	✓

Table 6: Characteristics of publications in telecare and homecare services (a)

References	Planning and decision level(s)	Forecasting purposes	Forecasting variables	Forecasting methods	Probabilistic forecasts
Setzler et al. [124]	Operational	To minimize response times to emergency calls	Emergency call volume	ANN,MA	✓
Veyron et al. [134]	Operational	To detect unplanned patient readmission	ED attendance	RF,LogR,LR	✗
Wong and Lai [146]	Operational	To manage ambulance fleet deployment	Emergency call volume	ARIMA	✗
Wooff and Stirling [147]	Operational	To optimize resources allocation and staff	Emergency call volume	LR,SLR,LWR	✓

Table 7: Characteristics of publications in telecare and homecare services (b)

References	Quality evaluation						Reproducibility	
	Benchmark	Out-of-sample evaluation	Evaluation metrics	Cross-validation	Uncertainty metrics	Business utility metrics	Data availability	Code availability
Al-Azzani et al. [6]	✓	✗	RMSE,MAPE,ME,MAE	✓	✗	✗	✗	✗
Argiento et al. [11]	✓	✓	MAE	✓	✓	✗	✗	✗
Brown et al. [28]	✗	✗	Average Peak Demand Rate,90 Ranked Demand	✓	✓	✗	✗	✗
Channouf et al. [34]	✓	✗	AIC,RMSE,MRAE	✓	✗	✗	✗	✗
Inouye et al. [65]	✗	✗	AUC-ROC,Chi-squared test,Hosmer-Lemeshow statistic	✓	✓	✗	✗	✗
Lanzarone et al. [85]	✓	✓	Error,RMSE,MAE	✓	✗	✓	✗	✗
Martin et al. [99]	✓	✓	MAPE,MAD	✓	✗	✗	✗	✗
Martinsson and Gustafsson [100]	✓	✗	WAIC,Error rate	✓	✓	✓	✗	✗
Matteson et al. [102]	✓	✓	RMSE	✓	✗	✗	✓	✓
Ordu et al. [110]	✓	✓	MASE	✓	✗	✗	✗	✗
Ordu et al. [111]	✓	✓	MASE	✓	✗	✓	✗	✗
Rostami-Tabar and Rendon-Sanchez [117]	✓	✓	MAE,ME,RMSE,Winkler score,Percentile score,CRPS	✓	✓	✗	✗	✗
Setzler et al. [124]	✓	✗	MSE	✓	✓	✗	✗	✗
Veyron et al. [134]	✓	✗	AUC-ROC	✓	✗	✗	✓	✗
Wong and Lai [146]	✓	✗	RMSE	✓	✗	✗	✗	✗
Wooff and Stirling [147]	✓	✗	Poisson-based errors,Approximate t-based errors	✓	✓	✗	✗	✗

Table 8: Characteristics of Publications in Ambulatory Care Services (a)

References	Planning and decision level(s)	Forecasting purposes	Forecasting variables	Forecasting methods	Probabilistic forecasts
Batal et al. [21]	Strategic,Operational	To detect unplanned patient readmissions	ED attendances	SLR	✓
Côté and Smith [38]	Operational	To optimize resource allocation and staffing	Radiology demand	LR	✗
DeLurgio et al. [41]	Tactical	To optimize resource allocation and staffing	Prehospital attendances	ARIMA, ETS, HW	✓
Demir [42]	Tactical	To detect unplanned patient readmission	Patient readmission	LogR, CT, GAMs, MARS	✗
García-Olmos et al. [50]	Strategic	To predict patients' risk of death	Patient readmission	LogR	✓
Heins et al. [61]	Strategic	To detect unplanned patient readmissions	Patient readmission, ED attendances	MPR	✗
Holleman et al. [64]	Operational	To optimize resource allocation and staffing	ED attendances	LR	✓
Inouye et al. [65]	Strategic	To detect unplanned patient readmission	Patient readmission	LogR	✓
Jalalpour et al. [66]	Tactical,Operational	To optimize resource allocation and staffing	ED attendances	GARMA	✓
Lee et al. [86]	Operational	To prevent inappropriate patient discharge	Healthcare utilizations	LogR, ANN, SVM	✗
Leeds et al. [87]	Operational	To detect unplanned patient readmissions, prevent inappropriate discharge	Patient readmission	CSM, LogR	✗

Table 8: Characteristics of Publications in Ambulatory Care Services (a)

References	Planning and decision level(s)	Forecasting purposes	Forecasting variables	Forecasting methods	Probabilistic forecasts
Martinsson and Gustafsson [100]	Tactical	To estimate the effects of telephone nursing	Healthcare utilizations	BOR	✓
McAuliffe et al. [105]	Operational	To detect unplanned patient readmissions	Patient readmission	LogR	✓
McRae [106]	Strategic	To optimize resource allocation and staffing	Regional demand for hospital services	ARIMA, ETS, ANN, LR	✗
Ordu et al. [110]	Operational,Tactical	To optimize resource allocation and staffing	Service demand	ARIMA, ETS, LR, STLF	✗
Ordu et al. [111]	Operational,Tactical	To optimize resource allocation and staffing	Service demand	SLR, ETS, STLF, ARIMA	✗
Stegink et al. [127]	Strategic	To identify reasons for ED overcrowding	Healthcare utilizations	LR, LogR	✗
Wooff and Stirling [147]	Operational	To optimize resource allocation and staffing	Emergency call volume	LR, SLR, LWR	✓
Yu et al. [152]	Tactical,Strategic	To detect unplanned patient readmissions	Prehospital attendances	ANN, WD, ETS	✗

Table 9: Characteristics of Publications in Ambulatory Care Services (b) - Continuation of Table (a)

References	Quality evaluation						Reproducibility	
	Benchmark	Out-of-sample evaluation	Evaluation metrics	Cross-validation	Uncertainty metrics	Business utility metrics	Data availability	Code availability
Batal et al. [21]	✗	✓	SBC, R-squared	✗	✓	✗	✗	✗
Côté and Smith [38]	✗	✓	RMSPE	✓	✗	✗	✗	✗
DeLurgio et al. [41]	✓	✓	RMSE, SAPE	✓	✓	✗	✗	✗
Demir [42]	✗	✓	AUC-ROC, BS, AIC, Sensitivity, Specificity, Precision, Recall	✓	✗	✗	✗	✗
García-Olmos et al. [50]	✓	✗	AUC-ROC, R-squared, BS, HLS	✓	✓	✗	✓	✗
Heins et al. [61]	✗	✗	AUC-ROC, R-squared, Sensitivity, PPV	✗	✗	✗	✓	✗
Holleman et al. [64]	✓	✓	R-squared, AUC-ROC	✗	✓	✗	✗	✗
Inouye et al. [65]	✗	✓	AUC-ROC, Chi-squared, HLS	✓	✓	✗	✗	✗
Jalalpour et al. [66]	✓	✓	RMSE, SS, Residual	✓	✓	✗	✗	✗
Lee et al. [86]	✗	✓	Sensitivity, Precision, AUC-ROC	✓	✗	✗	✗	✗
Leeds et al. [87]	✗	✓	AIC, AUC-ROC	✓	✗	✗	✗	✗
Martinsson and Gustafsson [100]	✓	✓	WAIC, ER	✓	✓	✓	✗	✗
McAuliffe et al. [105]	✓	✓	AUC-ROC, HLS	✓	✓	✗	✗	✗
McRae [106]	✗	✓	MSE, MAE, MAPE, MASE	✓	✗	✗	✗	✗
Ordu et al. [110]	✗	✓	MASE	✓	✗	✗	✗	✗
Ordu et al. [111]	✗	✓	MASE	✓	✓	✓	✗	✗
Stegink et al. [127]	✗	✗	R-squared	✗	✗	✗	✓	✗
Wooff and Stirling [147]	✓	✓	PBE, ATE	✓	✓	✗	✗	✗
Yu et al. [152]	✗	✓	RMSE, MAPE	✓	✗	✗	✗	✗

Table 10: Characteristics of Publications in Emergency Care Services (a)

References	Planning and decision level(s)	Forecasting purposes	Forecasting variables	Forecasting methods	Probabilistic forecasts
Aboagye-Sarfo et al. [1]	Strategic	To optimize resource allocation and staffing	ED attendances	V-ARMA, HW, ARMA	✗
Afilal et al. [4]	Operational, Tactical, Strategic	To optimize resource allocation and staffing	ED attendances	ARMA, AM	✗
Ahmed and Frohn [5]	Operational	To optimize resource allocation and staffing	ED attendances	RF	✗
Al-Azzani et al. [6]	Operational, Tactical	To minimize response times to emergency calls	Emergency call volume	ARIMA, HW, LR, SSA	✗
Ang et al. [8]	Operational	To improve ED patients' throughput time	Patient waiting time and LOS	MA, QR, Q-LASSO, FME	✓
Araz et al. [9]	Operational, Tactical	To optimize resource allocation and staffing	ED attendances	LR, ARIMA, HW, SLR	✓
Arora et al. [13]	Operational	To improve ED patients' throughput time	Patient waiting time and LOS	QR, Q-LASSO, KNN	✓
Aroua and Abdul-Nour [14]	Operational, Tactical	To improve ED patients' throughput time	ED attendances	ARIMA, LR, SARIMA	✓
Athanasopoulos et al. [17]	Operational, Tactical, Strategic	To introduce the concept of Temporal Hierarchies for time series forecasting	ED attendances	ETS, ARIMA	✓
Baas et al. [18]	Operational, Tactical	To forecast ICU occupancy	Bed occupancy	PR, MA, LMA	✓

Table 10: Characteristics of Publications in Emergency Care Services (a)

References	Planning and decision level(s)	Forecasting purposes	Forecasting variables	Forecasting methods	Probabilistic forecasts
Barros et al. [20]	Tactical	To optimize resource allocation and staffing	ED attendances	MA, LR, ANN, SVM	✓
Ben-Assuli and Padman [22]	Operational	To detect unplanned patient readmissions	Patient readmission	LogR, DT, SVM, CNN, BBM	✗
Bergs et al. [24]	Strategic	To optimize resource allocation and staffing	ED attendances	ETS, Naive	✓
Blair et al. [26]	Strategic, Tactical, Operational	To detect unplanned patient readmissions	ED attendances	PR	✗
Boyle et al. [27]	Operational, Tactical	To optimize resource allocation and staffing	ED attendances	LR, ARIMA, ETS, MA	✓
Calegari et al. [29]	Operational	To analyse the effects of climatic and calendrical factors	ED attendances	ETS, SMHW, SARIMA, MSARIMA	✗
Cameron et al. [30]	Operational	To improve ED patients' throughput time	Patient readmission	LogR	✓
Carvalho-Silva et al. [31]	Operational	To optimize resource allocation and staffing	ED attendances	ARIMA, MA, HW, ETS	✗
Champion et al. [33]	Tactical	To optimize resource allocation and staffing	ED attendances	ARIMA, ETS	✗
Choudhury and Urena [37]	Operational	To optimize resource allocation and staffing	ED attendances	ARIMA, HW, TBATS, ANN	✗

Table 10: Characteristics of Publications in Emergency Care Services (a)

References	Planning and decision level(s)	Forecasting purposes	Forecasting variables	Forecasting methods	Probabilistic forecasts
Cui et al. [39]	Operational	To detect unplanned patient readmissions, To provide support for discharge management, To prevent the inappropriate discharge of patients	Patient readmission	SVM, ARIMA, DT, NBT	✗
Demir [42]	Tactical	To detect unplanned patient readmission	Patient readmission	LogR, CT, GAMs, MARS	✗
Ekström et al. [46]	Operational	To optimize resource allocation and staffing	ED attendances	LR	✗
Gallagher et al. [49]	Operational	To detect unplanned patient readmissions	Patient readmission	LASSO	✓
Gartner and Padman [51]	Operational	To improve ED patients' throughput time	Patient waiting time and LOS	NBT, BN, DT, DRL	✗
Gillard and Knight [53]	Operational	To optimize resource allocation and staffing	Staff requirements	SSA	✗
Golmohammadi [54]	Operational	To detect unplanned patient readmissions	ED patient admissions, Patient readmission	LogR, ANN	✗
Grekousis and Liu [56]	Operational	To manage ambulance fleet deployment	ED attendances, Ambulance demand	ANN	✗
Guntuku et al. [58]	Operational	To optimize resource allocation and staffing	Patient readmission, ED attendances	RF, SVM, Catboost, LogR	✓
Hariman et al. [59]	Operational	To detect unplanned patient readmissions	Patient readmission	LogR	✓

Table 10: Characteristics of Publications in Emergency Care Services (a)

References	Planning and decision level(s)	Forecasting purposes	Forecasting variables	Forecasting methods	Probabilistic forecasts
Hertzum [63]	Operational	To improve ED patients' throughput time	ED attendances	ARIMA, LR, Naive	✓
Holleman et al. [64]	Operational	To optimize resource allocation and staffing	ED attendances	LR	✓
Jalalpour et al. [66]	Tactical, Operational	To optimize resource allocation and staffing	ED attendances	GARMA	✓
Jilani et al. [67]	Operational, Tactical	To optimize resource allocation and staffing	ED attendances	FTS, ARIMA, ANN	✓
Jones et al. [69]	Operational	To optimize resource allocation and staffing	Bed occupancy	SARIMA	✓
Jones et al. [70]	Operational	To optimize resource allocation and staffing	ED attendances	LR, SARIMA, ETS, ANN	✗
Jones et al. [71]	Operational	To optimize resource allocation and staffing	ED attendances	ETS, VAR	✗
Juang et al. [72]	Tactical, Strategic	To identify reasons for ED overcrowding	ED attendances	ARIMA	✗
Kadri et al. [74]	Operational	To optimize resource allocation and staffing	ED attendances	ARIMA	✗
Kam et al. [76]	Operational	To optimize resource allocation and staffing	ED attendances	MA, ARIMA, SARIMA	✗
King et al. [80]	Operational	To predict patients' risk of death	ED attendances	LogR	✗

Table 10: Characteristics of Publications in Emergency Care Services (a)

References	Planning and decision level(s)	Forecasting purposes	Forecasting variables	Forecasting methods	Probabilistic forecasts
Kirby et al. [81]	Strategic	To detect unplanned patient readmissions	ED attendances	LogR	✓
Klunder et al. [82]	Tactical	To detect unplanned patient readmissions	Patient readmission, ED return visits	LogR	✗
Kortbeek et al. [84]	Operational	To optimize resource allocation and staffing	Bed occupancy	GAA	✓
Lee et al. [86]	Operational	To prevent the inappropriate discharge of patients	Healthcare utilizations	LogR, ANN, SVM	✗
Lin et al. [88]	Operational	To manage ambulance fleet deployment	Ambulance demand	MA, LR, SVM, MLP, RBFN, LightGBM	✗
Lo et al. [92]	Operational	To detect unplanned patient readmissions	Patient readmission	RF, LogR, LGBM, Xgboost, Catboost	✗
Marcilio et al. [97]	Operational	To analyse the effects of climatic and calendrical factors	ED attendances	GLM, GEE, SARIMA	✓
Martinsson and Gustafsson [100]	Tactical	To estimate the effects of telephone nursing	Healthcare utilizations	BOR	✓
Matheny et al. [101]	Operational	To detect unplanned patient readmissions	Patient readmission	LASSO, RR, EN, RF, Catboost	✗
McAllan et al. [104]	Operational, Strategic	To optimize resource allocation and staffing	ED attendances	RW, NBR	✗
McRae [106]	Strategic	To optimize resource allocation and staffing	Regional demand for hospital services.	ARIMA, ETS, ANN, LR	✗
Mišić et al. [108]	Operational	To detect unplanned patient readmissions	Patient readmission	LogR	✗

Table 10: Characteristics of Publications in Emergency Care Services (a)

References	Planning and decision level(s)	Forecasting purposes	Forecasting variables	Forecasting methods	Probabilistic forecasts
Nijman et al. [109]	Operational	To detect unplanned patient readmission	Patient readmission, ED attendances	LogR	✓
Ordu et al. [110]	Operational, Tactical	To optimize resource allocation and staffing	Service demand	ARIMA, ETS, LR, STLF	✗
Ordu et al. [111]	Operational, Tactical	To optimize resource allocation and staffing	Service demand	SLR, ETS, STLF, ARIMA	✗
Pauly et al. [113]	Operational	To detect unplanned patient readmissions	Patient readmission	LogR	✓
Pellerin et al. [114]	Operational	To detect unplanned patient readmissions	ED return visits	LogR	✓
Rostami-Tabar and Ziel [118]	Operational, Tactical	To analyse the effects of climatic and calendrical factors	ED attendances	LASSO, RR, EN, ARIMA, ETS	✓
Sharmin et al. [125]	Operational	To detect unplanned patient readmissions	Patient readmission	LogR, LR	✓
Sudarshan et al. [128]	Operational	To detect unplanned patient readmissions	ED attendances	RF, LSTM, CNN	✗
Sun et al. [129]	Operational	To optimize resource allocation and staffing	ED attendances	ARIMA	✓
Veyron et al. [134]	Operational	To detect unplanned patient readmissions	ED attendances	RF, LogR, LR	✗
Vile et al. [135]	Operational	To manage ambulance fleet deployment	Ambulance demand	SSA, ARIMA, HW	✗
Vile et al. [136]	Operational	To manage ambulance fleet deployment	Ambulance demand	HW, ARIMA, SSA	✗

Table 10: Characteristics of Publications in Emergency Care Services (a)

References	Planning and decision level(s)	Forecasting purposes	Forecasting variables	Forecasting methods	Probabilistic forecasts
Villani et al. [137]	Strategic, Tactical	To optimize resource allocation and staffing	Prehospital attendances	ARIMA, SARIMA, ETS	✗
Walsh et al. [139]	Strategic	To detect unplanned patient readmissions	Patient readmission, Patient waiting time and LOS	LogR	✓
Wang et al. [140]	Operational, Tactical	To detect unplanned patient readmissions	Patient readmission	LogR, JF	✗
Whitt and Zhang [143]	Operational	To improve ED patients' throughput time	Patient waiting time and LOS	ARIMA, QM	✗
Whitt and Zhang [144]	Operational	To improve ED patients' throughput time	ED attendances	SARIMA, ANN, MLP	✗
Xu et al. [148]	Operational, Strategic	To optimize resource allocation and staffing	ED attendances	ANN, NLLSR, LR	✗
Xu et al. [149]	Operational	To analyse the effects of climatic and calendrical factors	ED attendances	ARIMA, ANN, GLM	✗
Yaghmaei et al. [151]	Operational	To detect unplanned patient readmissions	ED attendances	LogR, LGBM, GBT	✓
Yu et al. [152]	Tactical, Strategic	To detect unplanned patient readmissions	Prehospital attendances	ANN, WD, ETS	✗
Zibners et al. [155]	Operational	To optimize resource allocation and staffing	ED attendances	ARIMA, LR	✗

Table 11: Characteristics of Publications in Emergency Care Services (b) - Continuation of Table (a)

References	Quality evaluation						Reproducibility	
	Benchmark	Out-of-sample evaluation	Evaluation metrics	Cross-validation	Uncertainty metrics	Business utility metrics	Data availability	Code availability
Aboagye-Sarfo et al. [1]	✓	✓	MAE, MAPE, RMSE	✗	✗	✗	✗	✗
Afilal et al. [4]	✗	✓	RMSE, RMAP	✓	✗	✗	✗	✗
Ahmed and Frohn [5]	✗	✓	Accuracy, Precision, Recall, F1-score	✓	✗	✓	✗	✗
Al-Azzani et al. [6]	✓	✗	RMSE, MAPE, ME, MAE	✓	✗	✗	✗	✗
Ang et al. [8]	✓	✓	MSE	✓	✓	✓	✗	✓
Araz et al. [9]	✓	✓	RMSE	✓	✓	✗	✗	✗
Arora et al. [13]	✓	✓	CRPS, RMSE, MAE, RPS	✓	✓	✓	✗	✗
Aroua and Abdul-Nour [14]	✓	✓	MAPE, MAE, WMAPE, MSE	✓	✓	✗	✗	✗
Athanasopoulos et al. [17]	✓	✓	RMAE, MASE	✓	✓	✗	✓	✓
Baas et al. [18]	✓	✓	CI, MAE, CR	✓	✓	✓	✗	✓
Barros et al. [20]	✓	✓	MAPE, MSE	✓	✓	✓	✗	✗
Ben-Assuli and Padman [22]	✗	✓	AUC-ROC, Precision, Accuracy, F1-score, Recall	✗	✓	✗	✗	✗
Bergs et al. [24]	✓	✓	MAPE, MASE, MAE	✓	✓	✗	✗	✗
Blair et al. [26]	✗	✓	P-value	✗	✗	✗	✗	✗
Boyle et al. [27]	✓	✗	MAPE	✓	✓	✗	✓	✗
Calegari et al. [29]	✗	✓	MAPE	✓	✗	✗	✗	✗
Cameron et al. [30]	✓	✓	AUC-ROC	✓	✓	✗	✗	✗
Carvalho-Silva et al. [31]	✗	✓	MAPE	✓	✗	✗	✗	✗
Champion et al. [33]	✗	✓	RMSE	✗	✗	✗	✗	✗
Choudhury and Urena [37]	✗	✓	AIC, SBC, ME, RMSE	✓	✗	✗	✗	✗
Cui et al. [39]	✗	✓	F1-score, Recall, G-mean, AUC-ROC, Accuracy, Sensitivity	✓	✗	✗	✓	✗
Demir [42]	✓	✓	AUC-ROC, BS, AIC, Sensitivity, Specificity, Precision, Recall	✓	✗	✗	✗	✗
Ekström et al. [46]	✗	✓	MAPE	✓	✗	✗	✗	✗
Gallagher et al. [49]	✓	✗	AUC-ROC, PPV	✗	✗	✗	✗	✗
Gartner and Padman [51]	✗	✓	Accuracy, Precision, AUC-ROC	✓	✗	✗	✗	✗
Gillard and Knight [53]	✗	✓	WMSE, RMSE	✓	✗	✓	✗	✓
Golmohammadi [54]	✗	✓	MAE, MSE, Accuracy	✗	✗	✗	✗	✗

Table 11: Characteristics of Publications in Emergency Care Services (b) - Continuation of Table (a)

References	Quality evaluation						Reproducibility	
	Benchmark	Out-of-sample evaluation	Evaluation metrics	Cross-validation	Uncertainty metrics	Business utility metrics	Data availability	Code availability
Grekousis and Liu [56]	✗	✓	ME, MSE	✓	✗	✗	✗	✗
Guntuku et al. [58]	✓	✓	AUC-ROC, F1-score	✓	✓	✗	✗	✗
Hariman et al. [59]	✓	✗	AUC-ROC, Sensitivity, Specificity, PPV, NPV	✓	✓	✗	✗	✗
Hertzum [63]	✓	✓	MAE, MAPE, MASE	✓	✓	✓	✗	✓
Holleman et al. [64]	✓	✗	R-squared, AUC-ROC	✓	✗	✗	✗	✗
Jalalpour et al. [66]	✓	✓	RMSE, SS, Residual	✓	✓	✗	✗	✗
Jilani et al. [67]	✓	✓	RMSE, MAPE, AIC	✓	✓	✗	✗	✗
Jones et al. [69]	✓	✗	RMSE	✓	✓	✓	✗	✗
Juang et al. [72]	✗	✓	MAPE, AIC, SBC	✗	✗	✗	✗	✗
Kadri et al. [74]	✗	✓	RMSE, R-squared, MAPE, MAD, MSE, BIC	✗	✗	✗	✗	✗
Kam et al. [76]	✗	✓	MAPE, AIC, BIC	✗	✗	✗	✗	✗
King et al. [80]	✗	✗	AIC, AUC-ROC, OR	✗	✗	✗	✗	✗
Kirby et al. [81]	✓	✗	CI, R-squared	✗	✗	✗	✗	✗
Klunder et al. [82]	✗	✓	AUC-ROC	✗	✗	✗	✓	✗
Kortbeek et al. [84]	✓	✗	MAE, MAPE	✓	✓	✗	✗	✗
Lee et al. [86]	✓	✓	Sensitivity, Precision, AUC-ROC	✓	✓	✓	✗	✗
Lin et al. [88]	✓	✓	WAPE, MAE, MAPE	✗	✗	✗	✗	✗
Lo et al. [92]	✓	✓	Precision, Recall, F1-score, AUC-ROC, AUPRC	✓	✗	✗	✗	✗
Marcilio et al. [97]	✓	✓	MAPE	✓	✗	✗	✗	✗
Martinsson and Gustafsson [100]	✓	✓	WAIC, ER	✓	✗	✗	✗	✗
Matheny et al. [101]	✗	✓	Sensitivity, Specificity, PPV, NPV, F1-score, AUC-ROC	✓	✗	✗	✗	✗
McAllan et al. [104]	✗	✓	MAPE	✗	✗	✗	✗	✗
McRae [106]	✓	✓	MSE, MAE, MAPE, MASE	✓	✗	✗	✗	✗
Mišić et al. [108]	✗	✓	AUC-ROC	✓	✗	✗	✗	✓
Nijman et al. [109]	✓	✓	AUC-ROC	✓	✗	✗	✗	✗

Table 11: Characteristics of Publications in Emergency Care Services (b) - Continuation of Table (a)

References	Quality evaluation						Reproducibility	
	Benchmark	Out-of-sample evaluation	Evaluation metrics	Cross-validation	Uncertainty metrics	Business utility metrics	Data availability	Code availability
Ordu et al. [110]	✗	✓	MASE	✓	✗	✗	✗	✗
Ordu et al. [111]	✗	✓	MASE	✓	✓	✗	✗	✗
Pauly et al. [113]	✓	✓	AUC-ROC, F1-score, Sensitivity, Specificity, Accuracy, Precision	✓	✗	✗	✗	✗
Pellerin et al. [114]	✓	✓	AUC-ROC	✓	✗	✗	✗	✗
Rostami-Tabar and Ziel [118]	✓	✓	MAE, RMSE, PS, ES	✓	✓	✗	✓	✓
Sharmin et al. [125]	✓	✓	Sensitivity, Specificity, AUC-ROC	✓	✗	✗	✗	✗
Sudarshan et al. [128]	✓	✓	MAPE, MSE	✓	✗	✗	✗	✗
Sun et al. [129]	✓	✓	MAPE	✓	✓	✗	✗	✗
Veyron et al. [134]	✓	✓	AUC-ROC	✗	✗	✗	✓	✗
Vile et al. [135]	✓	✓	RMSE, SD	✓	✗	✗	✗	✗
Vile et al. [136]	✓	✓	RMSE	✓	✗	✗	✗	✗
Villani et al. [137]	✗	✓	MAE, MAPE, MSE	✗	✗	✗	✓	✗
Walsh et al. [139]	✓	✓	HRS	✓	✗	✗	✗	✗
Wang et al. [140]	✗	✗	AUC-ROC	✗	✗	✗	✗	✗
Whitt and Zhang [143]	✗	✓	None	✗	✗	✗	✓	✗
Whitt and Zhang [144]	✓	✓	MSE, MAPE	✓	✗	✗	✗	✗
Xu et al. [148]	✓	✓	MAPE, R-squared, MSE	✓	✗	✗	✗	✗
Xu et al. [149]	✓	✓	MAPE, RMSE	✓	✗	✗	✗	✗
Yaghmaei et al. [151]	✓	✓	AUC-ROC, Recall, PPV	✓	✗	✗	✗	✗
Yu et al. [152]	✗	✓	RMSE, MAPE	✓	✗	✗	✗	✗
Zibners et al. [155]	✗	✓	R-squared, SE	✗	✗	✗	✗	✗

Table 12: Characteristics of Publications in Inpatient Care Services (a)

References	Planning and decision level(s)	Forecasting purposes	Forecasting variables	Forecasting methods	Probabilistic forecasts
Abuhay et al. [2]	Tactical, Strategic	To increase the credibility and acceptance of patient flow simulation models	ED patient admissions	SM, ARIMA, Prophet, Xgboost	✗
Adogwa et al. [3]	Operational	To detect unplanned patient readmissions	Patient readmission	LogR	✗
Al Ghamdi and Alshammari [7]	Operational	To detect unplanned patient readmissions	Patient readmission	SVM, ANN, DT	✗
Ashfaq et al. [15]	Operational	To detect unplanned patient readmissions	Patient readmission	LSTM, ANN, GRU	✓
Baig et al. [19]	Operational	To detect unplanned patient readmissions	Patient readmission	Xgboost, RF, Adaboost	✗
Ben-Chetrit et al. [23]	Tactical, Operational	To detect unplanned patient readmission	Patient readmission	LogR	✗
Bertsimas et al. [25]	Operational	To optimize resource allocation and staffing	Patient waiting time and LOS, Healthcare utilizations	RF, GBT, CART, LogR, OT	✓
Casalini et al. [32]	Operational	To detect unplanned patient readmissions	Patient readmission	LogR	✓
Chen et al. [35]	Operational	To improve the hospital's ability to tackle pandemics, To optimize resource allocation and staffing	COVID-19 confirmed cases	CNN, SEIR, ARIMA, FNN	✗

Table 12: Characteristics of Publications in Inpatient Care Services (a)

References	Planning and decision level(s)	Forecasting purposes	Forecasting variables	Forecasting methods	Probabilistic forecasts
Cui et al. [39]	Operational	To detect unplanned patient readmissions, To provide support for discharge management, To prevent the inappropriate discharge of patients	Patient readmission	SVM, ARIMA, DT, NBT	✗
Demir [42]	Tactical	To detect unplanned patient readmission	Patient readmission	LogR, CT, GAMs, MARS	✗
Deschepper et al. [43]	Operational	To detect unplanned patient readmissions	Patient readmission	LR, LogR	✗
Dhalluin et al. [44]	Operational	To detect unplanned patient readmission	Patient readmission	LogR, Catboost, RF, ANN	✗
Earnest et al. [45]	Operational	To optimize resource allocation and staffing	Bed occupancy	ARIMA	✗
England et al. [47]	Operational	To optimize resource allocation and staffing	Patient readmission	ETS, SM	✗
Gallagher et al. [49]	Operational	To detect unplanned patient readmissions	Patient readmission	LASSO	✓
Gartner et al. [52]	Operational	To optimize resource allocation and staffing	Healthcare utilizations	NBT, BN, CT, VC, PA	✓
Golmohammadi [54]	Operational	To detect unplanned patient readmissions	ED patient admissions, Patient readmission	LogR, ANN	✗
Goudjerkan and Jayabalan [55]	Operational	To detect unplanned patient readmissions	Patient readmission	RF, CNN	✓

Table 12: Characteristics of Publications in Inpatient Care Services (a)

References	Planning and decision level(s)	Forecasting purposes	Forecasting variables	Forecasting methods	Probabilistic forecasts
Heins et al. [60]	Operational, Tactical, Strategic	To optimize resource allocation and staffing	Bed occupancy	MC	✗
Heppleston et al. [62]	Strategic, Operational, Tactical	To detect unplanned patient readmissions	Patient readmission, Mortality	LogR	✗
Johnson et al. [68]	Operational	To improve the hospital's ability to tackle pandemics	COVID-19 confirmed cases	ARIMA, ARIMAX, NAR, HW, Naive, MA	✗
Jones et al. [69]	Operational	To optimize resource allocation and staffing	Bed occupancy	SARIMA	✓
Jones et al. [71]	Operational	To optimize resource allocation and staffing	ED attendances	ETS, VAR	✗
Kalagara et al. [75]	Operational	To detect unplanned patient readmissions	Patient readmission	GBM, LogR, DT	✓
Karhade et al. [77]	Tactical	To detect unplanned patient readmissions	Patient readmission	NLP, Xgboost	✗
Khan and Gupta [78]	Tactical	To improve the hospital's ability to tackle pandemics	COVID-19 confirmed cases	NAR, ARIMA	✗
Kortbeek et al. [84]	Operational	To optimize resource allocation and staffing	Bed occupancy	GAA	✓
Lee et al. [86]	Operational	To prevent the inappropriate discharge of patients	Healthcare utilizations	LogR, ANN, SVM	✗
Littig and Isken [90]	Operational	To optimize resource allocation and staffing	Bed occupancy	LR	✗

Table 12: Characteristics of Publications in Inpatient Care Services (a)

References	Planning and decision level(s)	Forecasting purposes	Forecasting variables	Forecasting methods	Probabilistic forecasts
Maali et al. [93]	Operational, Tactical	To detect unplanned patient readmissions	Patient readmission	GBT, LogR	✗
Maltenfort et al. [94]	Operational	To detect unplanned patient readmissions	Patient readmission	LogR, GLM	✓
Manning et al. [96]	Operational	To detect unplanned patient readmissions	Patient readmission	LogR, RF, CART	✓
Marcusson et al. [98]	Strategic	To detect unplanned patient readmissions	Patient readmission	LogR, LASSO	✓
Matheny et al. [101]	Operational	To detect unplanned patient readmissions	Patient readmission	LASSO, RR, EN, RF, Catboost	✗
McAuliffe et al. [105]	Operational	To detect unplanned patient readmissions	Patient readmission	LogR	✓
McRae [106]	Strategic	To optimize resource allocation and staffing	Regional demand for hospital services.	ARIMA, ETS, ANN, LR	✗
Mišić et al. [108]	Operational	To detect unplanned patient readmissions	Patient readmission	LogR	✗
Ordu et al. [110]	Operational, Tactical	To optimize resource allocation and staffing	Service demand	ARIMA, ETS, LR, STLF	✗
Ordu et al. [111]	Operational, Tactical	To optimize resource allocation and staffing	Service demand	SLR, ETS, STLF, ARIMA	✗
Pauly et al. [113]	Operational	To detect unplanned patient readmissions	Patient readmission	LogR	✓
Rostami-Tabar et al. [119]	Operational	To improve the hospital's ability to tackle pandemics	COVID-19 confirmed cases	QR, ETS, ARIMA, LR, Prophet	✓

Table 12: Characteristics of Publications in Inpatient Care Services (a)

References	Planning and decision level(s)	Forecasting purposes	Forecasting variables	Forecasting methods	Probabilistic forecasts
Sbrana [120]	Operational	To improve the hospital's ability to tackle pandemics	COVID-19 confirmed cases	ILL, ARIMA, ETS	✓
Schiltz et al. [122]	Operational	To detect unplanned patient readmissions	Patient readmission	RF, CART, GLM	✓
Schipmann et al. [123]	Operational	To detect unplanned patient readmissions	Patient readmission	LogR	✓
Sharmin et al. [125]	Operational	To detect unplanned patient readmissions	Patient readmission	LogR, LR	✓
Shulan et al. [126]	Operational	To detect unplanned patient readmissions	Patient readmission	LogR	✗
Teja et al. [130]	Operational	To detect unplanned patient readmissions	Patient readmission	SLR, LogR	✓
Tey et al. [131]	Operational	To detect unplanned patient readmission	Patient readmission	ANN, CNN, NBT, RF, LogR, SVM	✓
Van Walraven et al. [133]	Operational	To predict patients' risk of death	Patient readmission	LogR	✓
Wong et al. [145]	Operational	To improve ED patients' throughput time, To detect unplanned patient readmissions	Patient readmission	GBT	✓
Zhou et al. [154]	Tactical	To detect unplanned patient readmissions	Patient readmission	RF, GBT, EN, LogR	✓

Table 13: Characteristics of Publications in Inpatient Care Services (b) - Continuation of Table (a)

References	Quality evaluation						Reproducibility	
	Benchmark	Out-of-sample evaluation	Evaluation metrics	Cross-validation	Uncertainty metrics	Business utility metrics	Data availability	Code availability
Abuhay et al. [2]	✓	✓	MAE, Accuracy	✗	✗	✗	✗	✗
Adogwa et al. [3]	✗	✗	P-value	✗	✗	✗	✗	✗
Al Ghamdi and Alshammari [7]	✓	✓	Accuracy, AUC-ROC	✓	✗	✗	✗	✗
Ashfaq et al. [15]	✓	✓	AUC-ROC, F1-score	✓	✓	✗	✓	✗
Baig et al. [19]	✓	✗	F1-score, AUC-ROC, PPV, NPV, Sensitivity	✗	✗	✗	✗	✗
Ben-Chetrit et al. [23]	✗	✓	AUC-ROC, Specificity, Sensitivity	✗	✗	✗	✗	✗
Bertsimas et al. [25]	✓	✓	AUC-ROC, MAE, MRE	✓	✓	✗	✓	✗
Casalini et al. [32]	✓	✗	Specificity, Sensitivity	✗	✗	✗	✗	✗
Chen et al. [35]	✓	✓	MAE, MAPE, RMSPE	✗	✗	✗	✓	✓
Cui et al. [39]	✓	✓	F1-score, Recall, G-mean, AUC-ROC, Accuracy, Sensitivity	✓	✗	✗	✓	✗
Demir [42]	✓	✓	AUC-ROC, BS, AIC, Sensitivity, Specificity, Precision, Recall	✓	✗	✗	✗	✗
Deschepper et al. [43]	✗	✗	AIC, P-value	✗	✗	✗	✗	✗
Dhalluin et al. [44]	✓	✓	AUC-ROC, Sensitivity, Specificity, PPV, NPV	✓	✗	✗	✗	✗
Earnest et al. [45]	✗	✓	MAPE	✗	✗	✗	✗	✗
England et al. [47]	✗	✓	MSE	✗	✗	✗	✓	✗
Gallagher et al. [49]	✓	✗	AUC-ROC, PPV	✗	✗	✗	✗	✗
Gartner et al. [52]	✓	✓	MAD, Accuracy	✓	✓	✗	✓	✓
Golmohammadi [54]	✗	✓	MAE, MSE, Accuracy	✗	✗	✗	✗	✗
Goudjerkan and Jayabalan [55]	✓	✓	AUC-ROC, MSE, Accuracy, Recall, Precision	✓	✗	✗	✗	✗
Heins et al. [60]	✗	✗	None	✗	✗	✗	✗	✗
Heppleston et al. [62]	✗	✗	AUC-ROC, OR	✗	✗	✗	✗	✗
Johnson et al. [68]	✓	✓	MASE, MAE	✓	✗	✗	✗	✗
Jones et al. [69]	✓	✗	RMSE	✓	✗	✗	✓	✗
Jones et al. [71]	✓	✓	MAE	✓	✗	✗	✗	✗

Table 13: Characteristics of Publications in Inpatient Care Services (b) - Continuation of Table (a)

References	Quality evaluation						Reproducibility	
	Benchmark	Out-of-sample evaluation	Evaluation metrics	Cross-validation	Uncertainty metrics	Business utility metrics	Data availability	Code availability
Kalagara et al. [75]	✓	✓	AUC-ROC, Accuracy, Sensitivity, Specificity	✓	✗	✗	✗	✗
Karhade et al. [77]	✗	✓	AUPRC, BS, AUC-ROC	✗	✗	✗	✗	✗
Khan and Gupta [78]	✓	✓	BIC, R-squared, RMSE	✓	✗	✗	✓	✗
Kortbeek et al. [84]	✓	✗	MAE, MAPE	✗	✗	✗	✓	✗
Lee et al. [86]	✓	✓	Sensitivity, Precision, AUC-ROC	✓	✗	✗	✗	✗
Littig and Isken [90]	✗	✓	SD	✗	✗	✗	✗	✗
Maali et al. [93]	✗	✓	AUC-ROC, Sensitivity, Specificity, PPV	✓	✗	✗	✗	✗
Maltenfort et al. [94]	✓	✓	AUC-ROC	✓	✗	✗	✗	✗
Manning et al. [96]	✓	✓	Sensitivity, Specificity, NPV, PPV	✓	✗	✗	✗	✗
Marcusson et al. [98]	✓	✓	AUC-ROC, Sensitivity, Specificity, PPV, NPV	✓	✗	✗	✗	✗
Matheny et al. [101]	✓	✓	Sensitivity, Specificity, PPV, NPV, F1-score, AUC-ROC	✓	✗	✗	✗	✗
McAuliffe et al. [105]	✓	✓	AUC-ROC, HLS	✓	✗	✗	✗	✗
McRae [106]	✓	✓	MSE, MAE, MAPE, MASE	✓	✗	✗	✗	✗
Mišić et al. [108]	✗	✓	AUC-ROC	✓	✗	✗	✗	✓
Ordu et al. [110]	✓	✓	MASE	✓	✗	✗	✗	✗
Ordu et al. [111]	✓	✓	MASE	✓	✗	✓	✗	✗
Pauly et al. [113]	✓	✓	AUC-ROC, F1-score, Sensitivity, Specificity, Accuracy, Precision	✓	✗	✗	✗	✗
Rostami-Tabar et al. [119]	✓	✓	RMSE, MAE, AIC, RPS	✓	✓	✗	✓	✗
Sbrana [120]	✓	✓	MASE, RMSE	✓	✓	✗	✓	✓
Schiltz et al. [122]	✓	✓	AUC-ROC, AIC	✓	✓	✗	✗	✗
Schipmann et al. [123]	✗	✗	CI, P-value, OR	✗	✗	✗	✗	✗
Sharmin et al. [125]	✓	✓	Sensitivity, Specificity, AUC-ROC	✓	✗	✗	✗	✗
Shulan et al. [126]	✗	✓	AUC-ROC	✓	✗	✗	✗	✓
Teja et al. [130]	✓	✓	AUC-ROC, BS	✓	✗	✗	✓	✗
Tey et al. [131]	✓	✓	AUC-ROC, Accuracy	✓	✗	✗	✓	✓
Van Walraven et al. [133]	✗	✓	AUC-ROC, HLS	✓	✗	✗	✗	✗

Table 13: Characteristics of Publications in Inpatient Care Services (b) - Continuation of Table (a)

References	Quality evaluation						Reproducibility	
	Benchmark	Out-of-sample evaluation	Evaluation metrics	Cross-validation	Uncertainty metrics	Business utility metrics	Data availability	Code availability
Wong et al. [145]	✓	✓	AUC-ROC, AUPRC	✓	✗	✗	✗	✗
Zhou et al. [154]	✓	✓	AUC-ROC, Sensitivity, Specificity	✓	✓	✗	✗	✗

Table 14: Characteristics of Publications in Surgical Care Services (a)

References	Planning and decision level(s)	Forecasting purposes	Forecasting variables	Forecasting methods	Probabilistic forecasts
Abuhay et al. [2]	Tactical, Strategic	To increase the credibility and acceptance of patient flow simulation models	ED patient admissions	SM, ARIMA, Prophet, Xgboost	✗
Baas et al. [18]	Operational, Tactical	To forecast ICU occupancy	Bed occupancy	PR, MA, LMA	✓
Bertsimas et al. [25]	Operational	To optimize resource allocation and staffing	Patient waiting time and LOS, Healthcare utilizations	RF, GBT, CART, LogR, OT	✓
Cheng et al. [36]	Operational	To improve ED patients' throughput time	ICU transfers	RF	✓
DeLurgio et al. [41]	Tactical	To optimize resource allocation and staffing	Prehospital attendances	ARIMA, ETS, HW	✓
Heins et al. [60]	Operational, Tactical, Strategic	To optimize resource allocation and staffing	Bed occupancy	MC	✗
Junqueira et al. [73]	Operational	To detect unplanned patient readmissions	Patient readmission	SVM, RF, MLP	✗
Koestler et al. [83]	Operational	To optimize resource allocation and staffing	ED attendances	PAR, LogR	✓
Kortbeek et al. [84]	Operational	To optimize resource allocation and staffing	Bed occupancy	GAA	✓
Lee et al. [86]	Operational	To prevent the inappropriate discharge of patients	Healthcare utilizations	LogR, ANN, SVM	✗
Leeds et al. [87]	Operational	To detect unplanned patient readmissions, To prevent the inappropriate discharge of patients	Patient readmission	CSM, LogR	✗
Lin et al. [89]	Operational	To provide support for discharge management	Patient readmission	LogR, RF, NBT, SVM, LSTM, CNN	✓
Littig and Isken [90]	Operational	To optimize resource allocation and staffing	Bed occupancy	LR	✗
Livingstone et al. [91]	Operational	To analyse the effects of climatic and calendrical factors	ED attendances	GAMs	✗

Table 14: Characteristics of Publications in Surgical Care Services (a)

References	Planning and decision level(s)	Forecasting purposes	Forecasting variables	Forecasting methods	Probabilistic forecasts
Manca et al. [95]	Operational	To improve the hospital's ability to tackle pandemics	Bed occupancy	EMG, Gompertz model, LogR	✗
Mišić et al. [107]	Operational	To detect unplanned patient readmissions	Patient readmission	LogR, RF, GBT, SVM, ANN	✗
Mišić et al. [108]	Operational	To detect unplanned patient readmissions	Patient readmission	LogR	✓
Ordu et al. [110]	Operational, Tactical	To optimize resource allocation and staffing	Service demand	ARIMA, ETS, LR, STLF	✗
Ordu et al. [111]	Operational, Tactical	To optimize resource allocation and staffing	Service demand	SLR, ETS, STLF, ARIMA	✗
Pagel et al. [112]	Operational, Tactical	To analyse the effects of climatic and calendrical factors	ED attendances	MA, LR, ARIMA, SARIMA	✗
Schiele et al. [121]	Operational	To forecast ICU occupancy	Bed occupancy	ANN, SVM, KNN, DTR	✗
van de Sande et al. [132]	Operational	To prevent the inappropriate discharge of patients	Healthcare utilizations	RF, LogR, GBM	✓
Weyh et al. [142]	Operational	To detect unplanned patient readmissions	Patient readmission	LogR	✓
Yaesoubi et al. [150]	Operational, Tactical	To improve the hospital's ability to tackle pandemics	COVID-19 confirmed cases	DT	✗
Zinouri et al. [156]	Operational, Tactical	To optimize resource allocation and staffing	Surgical case volumes	SARIMA	✗

Table 15: Characteristics of Publications in Surgical Care Services (b) - Continuation of Table (a)

References	Quality evaluation						Reproducibility	
	Benchmark	Out-of-sample evaluation	Evaluation metrics	Cross-validation	Uncertainty metrics	Business utility metrics	Data availability	Code availability
Abuhay et al. [2]	✓	✓	MAE, Accuracy	✗	✗	✗	✗	✗
Baas et al. [18]	✓	✓	CI, MAE, CR	✓	✓	✓	✗	✓
Bertsimas et al. [25]	✓	✓	AUC-ROC, MAE, MRE	✓	✓	✓	✓	✗
Cheng et al. [36]	✓	✓	Sensitivity, Specificity, Accuracy, AUC-ROC	✓	✗	✗	✗	✗
DeLurgio et al. [41]	✓	✓	RMSE, SAPE	✗	✓	✗	✗	✗
Heins et al. [60]	✗	✗	None	✗	✗	✗	✗	✗
Junqueira et al. [73]	✓	✓	AUC-ROC, Precision, Recall, Accuracy	✓	✗	✗	✗	✗
Koestler et al. [83]	✓	✓	MAPE, MSE	✓	✓	✗	✗	✗
Kortbeek et al. [84]	✓	✗	MAE, MAPE	✓	✗	✓	✗	✗
Lee et al. [86]	✓	✓	Sensitivity, Precision, AUC-ROC	✓	✗	✗	✗	✗
Leeds et al. [87]	✗	✓	AIC, AUC-ROC	✓	✗	✗	✗	✗
Lin et al. [89]	✓	✓	AUC-ROC, Sensitivity, Specificity	✓	✓	✗	✓	✓
Littig and Isken [90]	✓	✓	SD	✗	✗	✗	✗	✗
Livingstone et al. [91]	✗	✓	AIC, REML	✗	✗	✗	✗	✗
Manca et al. [95]	✓	✓	RMSE, MAE, MEDAE	✗	✗	✗	✓	✓
Mišić et al. [107]	✓	✓	AUC-ROC, BS, PPV, NPV	✓	✗	✗	✗	✗
Mišić et al. [108]	✓	✓	AUC-ROC	✓	✗	✗	✗	✓
Ordu et al. [110]	✓	✓	MASE	✓	✗	✗	✗	✗
Ordu et al. [111]	✓	✓	MASE	✓	✗	✓	✗	✗
Pagel et al. [112]	✗	✓	SE, SD	✗	✗	✗	✓	✗
Schiele et al. [121]	✓	✓	RMSE, MSE	✓	✗	✓	✗	✗
van de Sande et al. [132]	✓	✓	AUC-ROC, Sensitivity, Specificity, PPV, NPV	✓	✗	✗	✗	✗
Weyh et al. [142]	✓	✗	P-value, OR	✓	✗	✗	✗	✗
Yaesoubi et al. [150]	✓	✗	Accuracy, Sensitivity, Specificity	✗	✗	✗	✗	✗
Zinouri et al. [156]	✓	✓	MAPE	✗	✗	✗	✗	✗

References

[1] P. Aboagye-Sarfo, Q. Mai, F. M. Sanfilippo, D. B. Preen, L. M. Stewart and D. M. Fatovich. A comparison of multivariate and univariate time series approaches to modelling and forecasting emergency department demand in Western Australia. *Journal of Biomedical Informatics*, 57:62–73, 2015.

[2] T. M. Abuhay, S. Robinson, A. Mamuye and S. V. Kovalchuk. Machine learning integrated patient flow simulation: why and how? *Journal of Simulation*, pages 1–14, 2023.

[3] O. Adogwa, A. A. Elsamadicy, A. I. Mehta, R. A. Vasquez, J. Cheng, I. O. Karikari and C. A. Bagley. Association between baseline affective disorders and 30-day readmission rates in patients undergoing elective spine surgery. *World Neurosurgery*, 94:432–436, 2016.

[4] M. Afilal, F. Yalaoui, F. Dugardin, L. Amodeo, D. Laplanche and P. Blua. Forecasting the emergency department patients flow. *Journal of Medical Systems*, 40(7):1–18, 2016.

[5] A. Ahmed and E. Frohn. A predictive and prescriptive analytical framework for scheduling language medical interpreters. *Health Care Management Science*, 24:531–550, 2021.

[6] M. A. Al-Azzani, S. Davari and T. J. England. An empirical investigation of forecasting methods for ambulance calls – a case study. *Health Systems*, 10(4):268–285, 2021.

[7] H. Al Ghamdi and R. Alshammari. Predicting hospital readmission within thirty-days. *Journal of Medical Imaging and Health Informatics*, 7(3):696–703, 2017.

[8] E. Ang, S. Kwasnick, M. Bayati, E. L. Plambeck and M. Aratow. Accurate emergency department wait time prediction. *Manufacturing & Service Operations Management*, 18 (1):141–156, 2016.

[9] O. M. Araz, D. Bentley and R. L. Muelleman. Using Google Flu Trends data in forecasting influenza-like–illness related visits in Omaha, Nebraska. *The American Journal of Emergency Medicine*, 32(9):1016–1023, 2014.

[10] O. M. Araz, D. Olson and A. Ramirez-Nafarrate. Predictive analytics for hospital admissions from the emergency department using triage information. *International Journal of Production Economics*, 208:199–207, 2019.

[11] R. Argiento, A. Guglielmi, E. Lanzarone and I. Nawajah. A bayesian framework for describing and predicting the stochastic demand of home care patients. *Flexible Services and Manufacturing Journal*, 28(1):254–279, 2016.

[12] R. Aringhieri, M. E. Bruni, S. Khodaparasti and J. T. van Essen. Emergency medical services and beyond: Addressing new challenges through a wide literature review. *Computers & Operations Research*, 78:349–368, 2017.

[13] S. Arora, J. W. Taylor and H.-Y. Mak. Probabilistic forecasting of patient waiting times in an emergency department. *Manufacturing & Service Operations Management*, 2023.

[14] A. Aroua and G. Abdul-Nour. Forecast emergency room visits–a major diagnostic categories based approach. *International Journal of Metrology and Quality Engineering*, 6 (2):204, 2015.

[15] A. Ashfaq, A. Sant Anna, M. Lingman and S. Nowaczyk. Readmission prediction using deep learning on electronic health records. *Journal of Biomedical Informatics*, 97:103256, 2019.

[16] E. Aspland, D. Gartner and P. Harper. Clinical pathway modelling: a literature review. *Health Systems*, 10(1):1–23, 2021.

[17] G. Athanasopoulos, R. J. Hyndman, N. Kourentzes and F. Petropoulos. Forecasting with temporal hierarchies. *European Journal of Operational Research*, 262(1):60–74, 2017.

[18] S. Baas, S. Dijkstra, A. Braaksma, P. van Rooij, F. J. Snijders, L. Tiemessen and R. J. Boucherie. Real-time forecasting of COVID-19 bed occupancy in wards and Intensive Care Units. *Health Care Management Science*, 24(2):402–419, 2021.

[19] M. Baig, N. Hua, E. Zhang, R. Robinson, D. Armstrong, R. Whittaker, T. Robinson, F. Mirza and E. Ullah. Predicting patients at risk of 30-day unplanned hospital readmission. *Studies in Health Technology and Informatics*, 266:20–24, 2019.

[20] O. Barros, R. Weber and C. Reveco. Demand analysis and capacity management for hospital emergencies using advanced forecasting models and stochastic simulation. *Operations Research Perspectives*, 8:100208, 2021.

[21] H. Batal, J. Tench, S. McMillan, J. Adams and P. S. Mehler. Predicting patient visits to an urgent care clinic using calendar variables. *Academic Emergency Medicine*, 8(1): 48–53, 2001.

[22] O. Ben-Assuli and R. Padman. Analysing repeated hospital readmissions using data mining techniques. *Health Systems*, 7(2):120–134, 2018.

[23] E. Ben-Chetrit, C. Chen-Shuali, E. Zimran, G. Munter and G. Nesher. A simplified scoring tool for prediction of readmission in elderly patients hospitalized in internal medicine departments. *The Israel Medical Association Journal*, 14(12):752–6, 2012.

[24] J. Bergs, P. Heerinckx and S. Verelst. Knowing what to expect, forecasting monthly emergency department visits: A time-series analysis. *International Emergency Nursing*, 22(2):112–115, 2014.

[25] D. Bertsimas, J. Pauphilet, J. Stevens and M. Tandon. Predicting inpatient flow at a major hospital using interpretable analytics. *Manufacturing & Service Operations Management*, 2021.

[26] M. Blair, A. J. Poots, V. Lim, S. Hiles, G. Greenfield, C. Crehan, B. Kugler and C. Boreham. Preschool children who are frequent attenders in emergency departments: an observational study of associated demographics and clinical characteristics. *Archives of Disease in Childhood*, 103(1):19–23, 2018.

[27] J. Boyle, M. Jessup, J. Crilly, D. Green, J. Lind, M. Wallis, P. Miller and G. Fitzgerald. Predicting emergency department admissions. *Emergency Medicine Journal*, 29(5):358–365, 2012.

[28] L. H. Brown, E. B. Lerner, B. Larmon, T. LeGassick and M. Taigman. Are EMS call volume predictions based on demand pattern analysis accurate? *Prehospital Emergency Care*, 11(2):199–203, 2007.

[29] R. Calegari, F. S. Fogliatto, F. R. Lucini, J. Neyeloff, R. S. Kuchenbecker and B. D. Schaan. Forecasting daily volume and acuity of patients in the emergency department. *Computational and Mathematical Methods in Medicine*, 2016, 2016.

[30] A. Cameron, K. Rodgers, A. Ireland, R. Jamdar and G. A. McKay. A simple tool to predict admission at the time of triage. *Emergency Medicine Journal*, 32(3):174–179, 2015.

[31] M. Carvalho-Silva, M. T. T. Monteiro, F. de Sá-Soares and S. Dória-Nóbrega. Assessment of forecasting models for patients arrival at emergency department. *Operations Research for Health Care*, 18:112–118, 2018.

[32] F. Casalini, S. Salvetti, S. Memmini, E. Lucaccini, G. Massimetti, P. L. Lopalco and G. P. Privitera. Unplanned readmissions within 30 days after discharge: improving quality through easy prediction. *International Journal for Quality in Health Care*, 29 (2):256–261, 2017.

[33] R. Champion, L. D. Kinsman, G. A. Lee, K. A. Masman, E. A. May, T. M. Mills, M. D. Taylor, P. R. Thomas and R. J. Williams. Forecasting emergency department presentations. *Australian Health Review*, 31(1):83–90, 2007.

[34] N. Channouf, P. L'Ecuyer, A. Ingolfsson and A. N. Avramidis. The application of forecasting techniques to modeling emergency medical system calls in Calgary, Alberta. *Health Care Management Science*, 10(1):25–45, 2007.

[35] Z.-Y. Chen, M. Sun and X.-X. Han. Prediction-driven collaborative emergency medical resource allocation with deep learning and optimization. *Journal of the Operational Research Society*, 74(2):590–603, 2023.

[36] F.-Y. Cheng, H. Joshi, P. Tandon, R. Freeman, D. L. Reich, M. Mazumdar, R. Kohli-Seth, M. A. Levin, P. Timsina and A. Kia. Using machine learning to predict icu transfer in hospitalized covid-19 patients. *Journal of clinical medicine*, 9(6):1668, 2020.

[37] A. Choudhury and E. Urena. Forecasting hourly emergency department arrival using time series analysis. *British Journal of Healthcare Management*, 26(1):34–43, 2020.

[38] M. J. Côté and M. A. Smith. Forecasting the demand for radiology services. *Health Systems*, 7(2):79–88, 2018.

[39] S. Cui, D. Wang, Y. Wang, P.-W. Yu and Y. Jin. An improved support vector machine-based diabetic readmission prediction. *Computer Methods and Programs in Biomedicine*, 166:123–135, 2018.

[40] L. Daines, S. McLean, A. Buelo, S. Lewis, A. Sheikh and H. Pinnock. Systematic review of clinical prediction models to support the diagnosis of asthma in primary care. *NPJ Primary Care Respiratory Medicine*, 29(1):1–9, 2019.

[41] S. DeLurgio, B. Denton, R. L. Cabanela, S. Bruggeman, A. R. Williams, S. Ward, N. Groves and J. Osborn. Forecasting weekly outpatient demands at clinics within a large medical center. *Production and Inventory Management Journal*, 45(2):35–46, 2009.

[42] E. Demir. A decision support tool for predicting patients at risk of readmission: A comparison of classification trees, logistic regression, generalized additive models, and multivariate adaptive regression splines. *Decision Sciences*, 45(5):849–880, 2014.

[43] M. Deschepper, P. Vermeir, D. Vogelaers, J. Devulder and K. Eeckloo. Is pain at discharge a risk factor for unplanned hospital readmission? *Acta Clinica Belgica*, 72(2): 95–102, 2017.

[44] T. Dhalluin, A. Bannay, P. Lemordant, E. Sylvestre, E. Chazard, M. Cuggia and G. Bouzille. Comparison of unplanned 30-day readmission prediction models, based on hospital warehouse and demographic data. In *Digital Personalized Health and Medicine*, pages 547–551. IOS Press, 2020.

[45] A. Earnest, M. I. Chen, D. Ng and L. Y. Sin. Using autoregressive integrated moving average (ARIMA) models to predict and monitor the number of beds occupied during a SARS outbreak in a tertiary hospital in Singapore. *BMC Health Services Research*, 5 (1):1–8, 2005.

[46] A. Ekström, L. Kurland, N. Farrokhnia, M. Castrén and M. Nordberg. Forecasting emergency department visits using internet data. *Annals of emergency medicine*, 65(4): 436–442, 2015.

[47] T. England, D. Gartner, E. Ostler, P. Harper, D. Behrens, J. Boulton, D. Bull, C. Cordeaux, I. Jenkins, F. Lindsay et al. Near real-time bed modelling feasibility study. *Journal of Simulation*, 15(4):261–272, 2021.

[48] M. M. Fry. A systematic review of the impact of afterhours care models on emergency departments, ambulance and general practice services. *Australasian Emergency Nursing Journal*, 14(4):217–225, 2011.

[49] D. Gallagher, C. Zhao, A. Brucker, J. Massengill, P. Kramer, E. G. Poon and B. A. Goldstein. Implementation and continuous monitoring of an electronic health record embedded readmissions clinical decision support tool. *Journal of Personalized Medicine*, 10(3):103, 2020.

[50] L. García-Olmos, R. Aguilar, D. Lora, M. Carmona, A. Alberquilla, R. García-Caballero, L. Sánchez-Gómez and C. Group. Development of a predictive model of hospitalization in primary care patients with heart failure. *PLoS One*, 14(8):e0221434, 2019.

[51] D. Gartner and R. Padman. Machine learning for healthcare behavioural OR: Addressing waiting time perceptions in emergency care. *Journal of the Operational Research Society*, 71(7):1087–1101, 2020.

[52] D. Gartner, R. Kolisch, D. B. Neill and R. Padman. Machine learning approaches for early DRG classification and resource allocation. *INFORMS Journal on Computing*, 27 (4):718–734, 2015.

[53] J. Gillard and V. Knight. Using singular spectrum analysis to obtain staffing level requirements in emergency units. *Journal of the Operational Research Society*, 65(5): 735–746, 2014.

[54] D. Golmohammadi. Predicting hospital admissions to reduce emergency department boarding. *International Journal of Production Economics*, 182:535–544, 2016.

[55] T. Goudjerkan and M. Jayabalan. Predicting 30-day hospital readmission for diabetes patients using multilayer perception. *International Journal of Advanced Computer Science and Applications*, 10(2), 2019.

[56] G. Grekousis and Y. Liu. Where will the next emergency event occur? predicting ambulance demand in emergency medical services using artificial intelligence. *Computers, Environment and Urban Systems*, 76:110–122, 2019.

[57] M. Gul and E. Celik. An exhaustive review and analysis on applications of statistical forecasting in hospital emergency departments. *Health Systems*, 9(4):263–284, 2020.

[58] S. C. Guntuku, H. A. Schwartz, A. Kashyap, J. S. Gaulton, D. C. Stokes, D. A. Asch, L. H. Ungar and R. M. Merchant. Variability in language used on social media prior to hospital visits. *Scientific Reports*, 10(1):1–9, 2020.

[59] K. Hariman, J. Lam, S. K. Leung, S. S. Lui et al. Clinical risk model to predict 28-day unplanned readmission via the accident and emergency department after discharge from acute psychiatric units for patients with psychotic spectrum disorders. *BJPsych open*, 6(1), 2020.

[60] J. Heins, J. Schoenfelder, S. Heider, A. R. Heller and J. O. Brunner. A scalable forecasting framework to predict covid-19 hospital bed occupancy. *INFORMS Journal on Applied Analytics*, 52(6):508–523, 2022.

[61] M. Heins, J. Korevaar, F. Schellevis and M. Rijken. Identifying multimorbid patients with high care needs-a study based on electronic medical record data. *European Journal of General Practice*, 26(1):189–195, 2020.

[62] E. Heppleston, C. H. Fry, K. Kelly, B. Shepherd, R. Wright, G. Jones, J. Robin, P. Murray, D. Fluck and T. S. Han. LACE index predicts age-specific unplanned readmissions and mortality after hospital discharge. *Aging Clinical and Experimental Research*, 33 (4):1041–1048, 2021.

[63] M. Hertzum. Forecasting hourly patient visits in the emergency department to counteract crowding. *The Ergonomics Open Journal*, 10(1), 2017.

[64] D. R. Holleman, R. L. Bowling and C. Gathy. Predicting daily visits to a walk-in clinic and emergency department using calendar and weather data. *Journal of General Internal Medicine*, 11(4):237–239, 1996.

[65] S. K. Inouye, Y. Zhang, R. N. Jones, P. Shi, L. A. Cupples, H. N. Calderon and E. R. Marcantonio. Risk factors for hospitalization among community-dwelling primary care older patients: development and validation of a predictive model. *Medical Care*, 46(7): 726, 2008.

[66] M. Jalalpour, Y. Gel and S. Levin. Forecasting demand for health services: Development of a publicly available toolbox. *Operations Research for Health Care*, 5:1–9, 2015.

[67] T. Jilani, G. Housley, G. Figueredo, P.-S. Tang, J. Hatton and D. Shaw. Short and long term predictions of hospital emergency department attendances. *International Journal of Medical Informatics*, 129:167–174, 2019.

[68] M. R. Johnson, H. Naik, W. S. Chan, J. Greiner, M. Michaleski, D. Liu, B. Silvestre and I. P. McCarthy. Forecasting ward-level bed requirements to aid pandemic resource planning: Lessons learned and future directions. *Health Care Management Science*, pages 1–24, 2023.

[69] S. A. Jones, M. P. Joy and J. Pearson. Forecasting demand of emergency care. *Health Care Management Science*, 5(4):297–305, 2002.

[70] S. S. Jones, A. Thomas, R. S. Evans, S. J. Welch, P. J. Haug and G. L. Snow. Forecasting daily patient volumes in the emergency department. *Academic Emergency Medicine*, 15 (2):159–170, 2008.

[71] S. S. Jones, R. S. Evans, T. L. Allen, A. Thomas, P. J. Haug, S. J. Welch and G. L. Snow. A multivariate time series approach to modeling and forecasting demand in the emergency department. *Journal of Biomedical Informatics*, 42(1):123–139, 2009.

[72] W.-C. Juang, S.-J. Huang, F.-D. Huang, P.-W. Cheng and S.-R. Wann. Application of time series analysis in modelling and forecasting emergency department visits in a medical centre in Southern Taiwan. *BMJ Open*, 7(11):e018628, 2017.

[73] A. R. B. Junqueira, F. Mirza and M. M. Baig. A machine learning model for predicting icu readmissions and key risk factors: analysis from a longitudinal health records. *Health and Technology*, 9(3):297–309, 2019.

[74] F. Kadri, F. Harrou, S. Chaabane and C. Tahon. Time series modelling and forecasting of emergency department overcrowding. *Journal of Medical Systems*, 38(9):1–20, 2014.

[75] S. Kalagara, A. E. Eltorai, W. M. Durand, J. M. DePasse and A. H. Daniels. Machine learning modeling for predicting hospital readmission following lumbar laminectomy. *Journal of Neurosurgery: Spine*, 30(3):344–352, 2018.

[76] H. J. Kam, J. O. Sung and R. W. Park. Prediction of daily patient numbers for a regional emergency medical center using time series analysis. *Healthcare Informatics Research*, 16(3):158–165, 2010.

[77] A. V. Karhade, O. Lavoie-Gagne, N. Agaronnik, H. Ghaednia, A. K. Collins, D. Shin and J. H. Schwab. Natural language processing for prediction of readmission in posterior lumbar fusion patients: which free-text notes have the most utility? *The Spine Journal*, 22(2):272–277, 2022.

[78] F. M. Khan and R. Gupta. Arima and nar based prediction model for time series analysis of covid-19 cases in india. *Journal of Safety Science and Resilience*, 1(1):12–18, 2020.

[79] S. H. Kim, H. S. Choi, E. S. Jin, H. Choi, H. Lee, S.-H. Lee, C. Y. Lee, M. G. Lee and Y. Kim. Predicting severe outcomes using national early warning score (NEWS) in

patients identified by a rapid response system: a retrospective cohort study. *Scientific Reports*, 11(1):1–8, 2021.

[80] C. D. King, V. W. Joyce, C. C. Nash, R. J. Buonopane, A. D. Sossong and K. J. Ressler. Emergency department use following pediatric psychiatric hospitalization. *Psychiatric Services*, 70(7):613–616, 2019.

[81] S. E. Kirby, S. M. Dennis, U. W. Jayasinghe and M. F. Harris. Frequent emergency attenders: is there a better way? *Australian Health Review*, 35(4):462–467, 2011.

[82] J. H. Klunder, V. Bordonis, M. W. Heymans, H. G. van der Roest, A. Declercq, J. H. Smit, V. Garms-Homolova, P. V. Jónsson, H. Finne-Soveri, G. Onder et al. Predicting unplanned hospital visits in older home care recipients: a cross-country external validation study. *BMC Geriatrics*, 21(1):1–9, 2021.

[83] D. C. Koestler, H. Ombao and J. Bender. Ensemble-based methods for forecasting census in hospital units. *BMC Medical Research Methodology*, 13(1):1–12, 2013.

[84] N. Kortbeek, A. Braaksma, F. H. Smeenk, P. J. Bakker and R. J. Boucherie. Integral resource capacity planning for inpatient care services based on bed census predictions by hour. *Journal of the Operational Research Society*, 66:1061–1076, 2015.

[85] E. Lanzarone, A. Matta and G. Scaccabarozzi. A patient stochastic model to support human resource planning in home care. *Production Planning and Control*, 21(1):3–25, 2010.

[86] S.-Y. Lee, R. B. Chinnam, E. Dalkiran, S. Krupp and M. Nauss. Prediction of emergency department patient disposition decision for proactive resource allocation for admission. *Health Care Management Science*, 23:339–359, 2020.

[87] I. L. Leeds, V. Sadiraj, J. C. Cox, X. S. Gao, T. M. Pawlik, K. E. Schnier and J. F. Sweeney. Discharge decision-making after complex surgery: Surgeon behaviors compared to predictive modeling to reduce surgical readmissions. *The American Journal of Surgery*, 213(1):112–119, 2017.

[88] A. X. Lin, A. F. W. Ho, K. H. Cheong, Z. Li, W. Cai, M. L. Chee, Y. Y. Ng, X. Xiao and M. E. H. Ong. Leveraging machine learning techniques and engineering of multi-nature features for national daily regional ambulance demand prediction. *International Journal of Environmental Research and Public Health*, 17(11):4179, 2020.

[89] Y.-W. Lin, Y. Zhou, F. Faghri, M. J. Shaw and R. H. Campbell. Analysis and prediction of unplanned intensive care unit readmission using recurrent neural networks with long short-term memory. *PLoS One*, 14(7):e0218942, 2019.

[90] S. J. Littig and M. W. Isken. Short term hospital occupancy prediction. *Health Care Management Science*, 10(1):47–66, 2007.

[91] S. Livingstone, C. Pagel, Z. Shao, E. Randle and P. Ramnarayan. Modelling the association between weather and short-term demand for children intensive care transport services during winter in the south east of england. *Operations Research for Health Care*, 31:100327, 2021.

[92] Y.-T. Lo, J. C.-h. Liao, M.-H. Chen, C.-M. Chang and C.-T. Li. Predictive modeling for 14-day unplanned hospital readmission risk by using machine learning algorithms. *BMC Medical Informatics and Decision Making*, 21(1):1–11, 2021.

[93] Y. Maali, O. Perez-Concha, E. Coiera, D. Roffe, R. O. Day and B. Gallego. Predicting 7-day, 30-day and 60-day all-cause unplanned readmission: a case study of a sydney hospital. *BMC Medical Informatics and Decision Making*, 18(1):1–11, 2018.

[94] M. G. Maltenfort, Y. Chen and C. B. Forrest. Prediction of 30-day pediatric unplanned hospitalizations using the johns hopkins adjusted clinical groups risk adjustment system. *PLoS One*, 14(8):e0221233, 2019.

[95] D. Manca, D. Caldiroli and E. Storti. A simplified math approach to predict icu beds and mortality rate for hospital emergency planning under covid-19 pandemic. *Computers & Chemical Engineering*, 140:106945, 2020.

[96] A. M. Manning, K. A. Casper, K. S. Peter, K. M. Wilson, J. R. Mark and R. M. Collar. Can predictive modeling identify head and neck oncology patients at risk for readmission? *Otolaryngology–Head and Neck Surgery*, 159(4):669–674, 2018.

[97] I. Marcilio, S. Hajat and N. Gouveia. Forecasting daily emergency department visits using calendar variables and ambient temperature readings. *Academic Emergency Medicine*, 20(8):769–777, 2013.

[98] J. Marcusson, M. Nord, H.-J. Dong and J. Lyth. Clinically useful prediction of hospital admissions in an older population. *BMC Geriatrics*, 20(1):1–9, 2020.

[99] R. J. Martin, R. Mousavi and C. Saydam. Predicting emergency medical service call demand: A modern spatiotemporal machine learning approach. *Operations Research for Health Care*, 28:100285, 2021.

[100] J. Martinsson and S. Gustafsson. Modeling the effects of telephone nursing on healthcare utilization. *International Journal of Medical Informatics*, 113:98–105, 2018.

[101] M. E. Matheny, I. Ricket, C. A. Goodrich, R. U. Shah, M. E. Stabler, A. M. Perkins, C. Dorn, J. Denton, B. E. Bray, R. Gouripeddi et al. Development of electronic health record–based prediction models for 30-day readmission risk among patients hospitalized for acute myocardial infarction. *JAMA Network Open*, 4(1):e2035782–e2035782, 2021.

[102] D. S. Matteson, M. W. McLean, D. B. Woodard and S. G. Henderson. Forecasting emergency medical service call arrival rates. *The Annals of Applied Statistics*, 5(2B): 1379–1406, 2011.

[103] J. H. May, W. E. Spangler, D. P. Strum and L. G. Vargas. The surgical scheduling problem: Current research and future opportunities. *Production and Operations Management*, 20(3):392–405, 2011.

[104] F. J. McAllan, D. Egerton-Warburton, G. O'Reilly, T. J. Weiland and G. A. Jelinek. Planning for the future: Modelling daily emergency department presentations in an australian capital city. *Emergency Medicine Australasia*, 31(5):750–755, 2019.

[105] L. McAuliffe, A. R. Zullo, R. Dapaah-Afriyie and C. Berard-Collins. Development and validation of a transitions-of-care pharmacist tool to predict potentially avoidable 30-day readmissions. *The Bulletin of the American Society of Hospital Pharmacists*, 75(3): 111–119, 2018.

[106] S. McRae. Long-term forecasting of regional demand for hospital services. *Operations Research for Health Care*, 28:100289, 2021.

[107] V. V. Mišić, E. Gabel, I. Hofer, K. Rajaram and A. Mahajan. Machine learning prediction of postoperative emergency department hospital readmission. *Anesthesiology*, 132 (5):968–980, 2020.

[108] V. V. Mišić, K. Rajaram and E. Gabel. A simulation-based evaluation of machine learning models for clinical decision support: application and analysis using hospital readmission. *NPJ Digital Medicine*, 4(1):1–11, 2021.

[109] R. G. Nijman, D. H. Borensztajn, J. M. Zachariasse, C. Hajema, P. Freitas, S. Greber-Platzer, F. J. Smit, C. F. Alves, J. van Der Lei, E. W. Steyerberg et al. A clinical prediction model to identify children at risk for revisits with serious illness to the emergency department: A prospective multicentre observational study. *PLoS One*, 16(7): e0254366, 2021.

[110] M. Ordu, E. Demir and C. Tofallis. A comprehensive modelling framework to forecast the demand for all hospital services. *The International Journal of Health Planning and Management*, 34(2):e1257–e1271, 2019.

[111] M. Ordu, E. Demir, C. Tofallis and M. M. Gunal. A novel healthcare resource allocation decision support tool: A forecasting-simulation-optimization approach. *Journal of the operational research society*, 72(3):485–500, 2021.

[112] C. Pagel, P. Ramnarayan, S. Ray and M. J. Peters. Development and implementation of a real time statistical control method to identify the start and end of the winter surge in demand for paediatric intensive care. *European Journal of Operational Research*, 264 (3):847–858, 2018.

[113] V. Pauly, H. Mendizabal, S. Gentile, P. Auquier and L. Boyer. Predictive risk score for unplanned 30-day rehospitalizations in the french universal health care system based on a medico-administrative database. *PLoS One*, 14(3):e0210714, 2019.

[114] G. Pellerin, K. Gao and L. Kaminsky. Predicting 72-hour emergency department revisits. *The American Journal of Emergency Medicine*, 36(3):420–424, 2018.

[115] N. C. Proudlove, S. Black and A. Fletcher. OR and the challenge to improve the NHS: modelling for insight and improvement in in-patient flows. *Journal of the Operational Research Society*, 58(2):145–158, 2007.

[116] S. Ramlakhan, S. Mason, C. O'Keeffe, A. Ramtahal and S. Ablard. Primary care services located with EDs: a review of effectiveness. *Emergency Medicine Journal*, 33(7):495–503, 2016.

[117] B. Rostami-Tabar and J. F. Rendon-Sanchez. Forecasting COVID-19 daily cases using phone call data. *Applied Soft Computing*, 100:106932, 2021.

[118] B. Rostami-Tabar and F. Ziel. Anticipating special events in emergency department forecasting. *International Journal of Forecasting*, 38(3):1197–1213, 2020.

[119] B. Rostami-Tabar, S. Arora, R.-S. Juan F and A. Goltsos. Probabilistic forecasting of daily covid-19 admissions using machine learning. *IMA Journal of Management Mathematics*, 2023.

[120] G. Sbrana. Modelling intermittent time series and forecasting covid-19 spread in the usa. *Journal of the Operational Research Society*, 74(2):465–475, 2023.

[121] J. Schiele, T. Koperna and J. O. Brunner. Predicting intensive care unit bed occupancy for integrated operating room scheduling via neural networks. *Naval Research Logistics (NRL)*, 68(1):65–88, 2021.

[122] N. K. Schiltz, M. A. Dolansky, D. F. Warner, K. C. Stange, S. Gravenstein and S. M. Koroukian. Impact of instrumental activities of daily living limitations on hospital readmission: an observational study using machine learning. *Journal of General Internal Medicine*, 35(10):2865–2872, 2020.

[123] S. Schipmann, E. Suero Molina, J. Windheuser, J. Doods, M. Schwake, E. Wilbers, S. Z. Alsofy, N. Warneke and W. Stummer. The 30-day readmission rate in neurosurgery – a useful indicator for quality assessment? *Acta Neurochirurgica*, 162(11):2659–2669, 2020.

[124] H. Setzler, C. Saydam and S. Park. EMS call volume predictions: A comparative study. *Computers & Operations Research*, 36(6):1843–1851, 2009.

[125] S. Sharmin, J. J. Meij, J. D. Zajac, A. R. Moodie and A. B. Maier. Predicting all-cause unplanned readmission within 30 days of discharge using electronic medical record data: A multi-centre study. *International Journal of Clinical Practice*, 75(8):e14306, 2021.

[126] M. Shulan, K. Gao and C. D. Moore. Predicting 30-day all-cause hospital readmissions. *Health Care Management Science*, 16:167–175, 2013.

[127] S. Stegink, A. M. Elliott and C. Burton. Statistical complexity of reasons for encounter in high users of out of hours primary care: analysis of a national service. *BMC Health Services Research*, 19(1):1–10, 2019.

[128] V. K. Sudarshan, M. Brabrand, T. M. Range and U. K. Wiil. Performance evaluation of emergency department patient arrivals forecasting models by including meteorological and calendar information: A comparative study. *Computers in Biology and Medicine*, 135:104541, 2021.

[129] Y. Sun, B. H. Heng, Y. T. Seow and E. Seow. Forecasting daily attendances at an emergency department to aid resource planning. *BMC Emergency Medicine*, 9(1):1–9, 2009.

[130] B. Teja, D. Raub, S. Friedrich, P. Rostin, M. D. Patrocínio, J. C. Schneider, C. Shen, G. A. Brat, T. T. Houle, R. W. Yeh et al. Incidence, prediction, and causes of unplanned 30-day hospital admission after ambulatory procedures. *Anesthesia & Analgesia*, 131 (2):497–507, 2019.

[131] S.-F. Tey, C.-F. Liu, T.-W. Chien, C.-W. Hsu, K.-C. Chan, C.-J. Chen, T.-J. Cheng and W.-S. Wu. Predicting the 14-day hospital readmission of patients with pneumonia using Artificial Neural Networks (ANN). *International Journal of Environmental Research and Public Health*, 18(10):5110, 2021.

[132] D. van de Sande, M. E. van Genderen, C. Verhoef, J. van Bommel, D. Gommers, E. van Unen, J. Huiskens and D. Grnhagen. Predicting need for hospital-specific interventional care after surgery using electronic health record data. *Surgery*, 170(3):790–796, 2021.

[133] C. Van Walraven, I. A. Dhalla, C. Bell, E. Etchells, I. G. Stiell, K. Zarnke, P. C. Austin and A. J. Forster. Derivation and validation of an index to predict early death or unplanned readmission after discharge from hospital to the community. *Canadian Medical Association Journal*, 182(6):551–557, 2010.

[134] J.-H. Veyron, P. Friocourt, O. Jeanjean, L. Luquel, N. Bonifas, F. Denis and J. Belmin. Home care aides observations and machine learning algorithms for the prediction of visits to emergency departments by older community dwelling individuals receiving home care assistance: A proof of concept study. *PLoS One*, 14(8):e0220002, 2019.

[135] J. L. Vile, J. W. Gillard, P. R. Harper and V. A. Knight. Predicting ambulance demand using singular spectrum analysis. *Journal of the Operational Research Society*, 63(11): 1556–1565, 2012.

[136] J. L. Vile, J. W. Gillard, P. R. Harper and V. A. Knight. Time-dependent stochastic methods for managing and scheduling Emergency Medical Services. *Operations Research for Health Care*, 8:42–52, 2016.

[137] M. Villani, A. Earnest, N. Nanayakkara, K. Smith, B. De Courten and S. Zoungas. Time series modelling to forecast prehospital EMS demand for diabetic emergencies. *BMC Health Services Research*, 17(1):1–9, 2017.

[138] E. Wallace, E. Stuart, N. Vaughan, K. Bennett, T. Fahey and S. M. Smith. Risk prediction models to predict emergency hospital admission in community-dwelling adults: a systematic review. *Medical Care*, 52(8):751, 2014.

[139] B. Walsh, H. C. Roberts and P. G. Nicholls. Features and outcomes of unplanned hospital admissions of older people due to ill-defined (R-coded) conditions: retrospective analysis of hospital admissions data in England. *BMC Geriatrics*, 11(1):1–7, 2011.

[140] H. Wang, R. D. Robinson, C. Johnson, N. R. Zenarosa, R. D. Jayswal, J. Keithley and K. A. Delaney. Using the LACE index to predict hospital readmissions in congestive heart failure patients. *BMC Cardiovascular Disorders*, 14(1):1–8, 2014.

[141] M. Wargon, B. Guidet, T. Hoang and G. Hejblum. A systematic review of models for forecasting the number of emergency department visits. *Emergency Medicine Journal*, 26(6):395–399, 2009.

[142] A. Weyh, R. Nocella, M. Abdelmalik, R. Pucci, A. Quimby, A. Bunnell and R. Fernandes. An analysis of unplanned readmissions after head and neck microvascular reconstructive surgery. *International Journal of Oral and Maxillofacial Surgery*, 49(12): 1559–1565, 2020.

[143] W. Whitt and X. Zhang. A data-driven model of an emergency department. *Operations Research for Health Care*, 12:1–15, 2017.

[144] W. Whitt and X. Zhang. Forecasting arrivals and occupancy levels in an emergency department. *Operations Research for Health Care*, 21:1–18, 2019.

[145] C. W. Wong, C. Chen, L. A. Rossi, M. Abila, J. Munu, R. Nakamura and Z. Eftekhari. Explainable tree-based predictions for unplanned 30-day readmission of patients with cancer using clinical embeddings. *JCO Clinical Cancer Informatics*, 5:155–167, 2021.

[146] H.-T. Wong and P.-C. Lai. Weather factors in the short-term forecasting of daily ambulance calls. *International Journal of Biometeorology*, 58(5):669–678, 2014.

[147] D. A. Wooff and S. G. Stirling. Practical statistical methods for call centres with a case study addressing urgent medical care delivery. *Annals of Operations Research*, 233(1): 501–515, 2015.

[148] M. Xu, T.-C. Wong and K.-S. Chin. Modeling daily patient arrivals at emergency department and quantifying the relative importance of contributing variables using artificial neural network. *Decision Support Systems*, 54(3):1488–1498, 2013.

[149] Q. Xu, K.-L. Tsui, W. Jiang and H. Guo. A hybrid approach for forecasting patient visits in emergency department. *Quality and Reliability Engineering International*, 32 (8):2751–2759, 2016.

[150] R. Yaesoubi, S. You, Q. Xi, N. A. Menzies, A. Tuite, Y. H. Grad and J. A. Salomon. Generating simple classification rules to predict local surges in covid-19 hospitalizations. *Health Care Management Science*, pages 1–12, 2023.

[151] E. Yaghmaei, L. Ehwerhemuepha, W. Feaster, D. Gibbs and C. Rakovski. A multicenter mixed-effects model for inference and prediction of 72-h return visits to the emergency department for adult patients with trauma-related diagnoses. *Journal of Orthopaedic Surgery and Research*, 15(1):1–12, 2020.

[152] L. Yu, G. Hang, L. Tang, Y. Zhao and K. Lai. Forecasting patient visits to hospitals using a WD&ANN-based decomposition and ensemble model. *Eurasia Journal of Mathematics, Science and Technology Education*, 13(12):7615–7627, 2017.

[153] H. Zhou, P. R. Della, P. Roberts, L. Goh and S. S. Dhaliwal. Utility of models to predict 28-day or 30-day unplanned hospital readmissions: an updated systematic review. *BMJ Open*, 6(6):e011060, 2016.

[154] H. Zhou, M. A. Albrecht, P. A. Roberts, P. Porter and P. R. Della. Using machine learning to predict paediatric 30-day unplanned hospital readmissions: a case-control retrospective analysis of medical records, including written discharge documentation. *Australian Health Review*, 45(3):328–337, 2021.

[155] L. M. Zibners, B. K. Bonsu, J. R. Hayes and D. M. Cohen. Local weather effects on emergency department visits: a time series and regression analysis. *Pediatric Emergency Care*, 22(2):104–106, 2006.

[156] N. Zinouri, K. M. Taaffe and D. M. Neyens. Modelling and forecasting daily surgical case volume using time series analysis. *Health Systems*, 7(2):111–119, 2018.
